# Foliation adjunction

**DOI:** 10.1007/s00208-024-03067-5

**Published:** 2024-12-21

**Authors:** Paolo Cascini, Calum Spicer

**Affiliations:** 1 Department of Mathematics, Imperial College London, 180 Queen’s Gate, London, SW7 2AZ UK; 2 Department of Mathematics, King’s College London, Strand, London, WC2R 2LS UK

**Keywords:** 14E30, 37F75

## Abstract

We present an adjunction formula for foliations on varieties and we consider applications of the adjunction formula to the cone theorem for rank one foliations and the study of foliation singularities.

## Introduction

Our primary goal is to present an adjunction formula for foliations on varieties defined over a field of characteristic zero.

Various special cases of the adjunction formula have appeared in [2, 3, 8, 10, 11, 29, 34] and [1]. We will give a treatment of the adjunction formula which unifies these other cases, and which is in line with the treatment of the adjunction formula for varieties (see for instance [26]).

Recall that the adjunction formula for varieties relates the canonical divisor of a smooth variety *X* and the canonical divisor of a smooth codimension one subvariety $D \subset X$ by the linear equivalence

$$\begin{aligned} (K_X+D)\vert _D \sim K_D. \end{aligned}$$

Given a foliation ${\mathcal {F}}$ of rank *r* on a smooth variety *X* and a smooth codimension one subvariety $D \subset X$ we can define a restricted foliation ${\mathcal {F}}_D$ on *D*. Roughly speaking, the leaves of ${\mathcal {F}}_D$ are the leaves of ${\mathcal {F}}$ intersected with *D*. Thus, ${\mathcal {F}}_D$ is a foliation of rank $r-\epsilon (D)$ where $\epsilon (D) = 1$ if *D* is not invariant by ${\mathcal {F}}$ and $\epsilon (D) = 0$ otherwise. The adjunction formula for foliations relates the divisors $(K_{\mathcal F}+\epsilon (D)D)\vert _D$ and $K_{{\mathcal {F}}_D}$. Even if *X*, *D* and ${\mathcal {F}}$ are all smooth, these two divisors will not in general be linearly equivalent. In analogy with the adjunction formula for singular varieties (e.g. see [24, §16]), we introduce a correction term $\textrm{Diff}_D({\mathcal {F}}) \ge 0$, called the different. With this correction term, we have a linear equivalence

$$\begin{aligned} (K_{{\mathcal {F}}}+\epsilon (D)D)\vert _D \sim K_{{\mathcal {F}}_D}+\textrm{Diff}_D({\mathcal {F}}). \end{aligned}$$

We refer to Proposition-Definition 3.7 for the definition and construction of the different. Note that a similar statement appeared in [3, Definition 3.11] in the case that *D* is invariant by ${{\mathcal {F}}}$.

In the case where *X* is a smooth surface and *C* is a smooth curve, this formula has been discussed in [8] in slightly different terms. When *C* is invariant by ${\mathcal {F}}$ the restricted foliation is the foliation with one leaf and so $K_{{\mathcal {F}}_C} = K_C$. In this case we have by [8, Proposition 2.3]

$$\begin{aligned} K_{{\mathcal {F}}}\vert _C \sim K_{{\mathcal {F}}_C}+Z({\mathcal {F}}, C) \end{aligned}$$

where $Z({\mathcal {F}}, C)$ depends on the singularities of ${\mathcal {F}}$ along *C*. When *C* is not invariant by ${\mathcal {F}}$ the restricted foliation is the foliation by points and so $K_{{\mathcal {F}}_C} = 0$. In this case we have by [8, Proposition 2.2]

$$\begin{aligned} (K_{{\mathcal {F}}}+C)\vert _C \sim K_{{\mathcal {F}}_C}+\textrm{tang}({\mathcal {F}}, C) \end{aligned}$$

where $\textrm{tang}({\mathcal {F}}, C)$ is the tangency divisor between ${\mathcal {F}}$ and *C*.

We will see that the correction term, $Z({\mathcal {F}}, C)$ or $\textrm{tang}({\mathcal {F}}, C)$, in each of these cases is equal to the different $\textrm{Diff}_C({\mathcal {F}})$. Assuming that *X* and *D* are possibly singular and that $S\rightarrow D$ is the normalisation map, we will explain the construction of the restricted foliation $\mathcal F_S$ on *S* and the induced different, see Proposition-Definition 3.7. We will also calculate the different in many cases, see Proposition 3.14.

Another important feature of the adjunction formula is that it gives a way to relate the singularities of ${\mathcal {F}}$ to ${\mathcal {F}}_D$. As an example of this type of statement we have the following:

### Theorem 1.1

(cf. Theorem 3.16) Let *X* be a ${\mathbb {Q}}$-factorial variety, let ${\mathcal {F}}$ be a foliation of rank *r* and let *D* be a prime divisor which is not ${\mathcal {F}}$-invariant. Suppose that $({\mathcal {F}}, D)$ is canonical (resp. log canonical). Let $S \rightarrow D$ be the normalisation of *D*.

Then $({\mathcal {F}}_S, \textrm{Diff}_S({{\mathcal {F}}}))$ is canonical (resp. log canonical).

As examples show, see Sect. 3.5.1, the non-invariance of *D* in the above statement is necessary.

Finally, we turn to some applications of the adjunction formula. Our first application is a proof of the cone theorem for pairs $({\mathcal {F}}, \Delta )$ where ${\mathcal {F}}$ is a rank one foliation (see also [7, Corollary IV.2.1] and [28]):

### Theorem 1.2

(= Theorem 4.8) Let *X* be a normal projective variety, let ${{\mathcal {F}}}$ be a rank one foliation and let $\Delta \ge 0$ so that $K_{{\mathcal {F}}}$ and $\Delta$ are ${\mathbb {Q}}$-Cartier and $({\mathcal {F}}, \Delta )$ is log canonical.

Then there are ${\mathcal {F}}$-invariant rational curves $C_1,C_2,\dots$ such that

$$\begin{aligned} 0<-(K_{{{\mathcal {F}}}}+\Delta )\cdot C_i\le 2\dim X \end{aligned}$$

and

$$\begin{aligned} \overline{\textrm{NE}}(X)=\overline{\textrm{NE}}(X)_{K_{{{\mathcal {F}}}}+\Delta \ge 0}+ \sum _i {\mathbb {R}}_+[C_i]. \end{aligned}$$

In [13, Theorem 1.2] a dynamical characterisation of ample line bundles on smooth surfaces was provided. As a consequence of the above theorem we are able to extend this to higher dimensions.

### Theorem 1.3

(= Corollary 4.10) Let *X* be a normal projective variety and let *L* be a $\mathbb {Q}$-Cartier divisor. Suppose that

1. $$L^{\dim X} \ne 0$$;
2. for some $q \in {\mathbb {Q}}_{>0}$ there exists a rank one foliation ${\mathcal {F}}$ with $K_{{\mathcal {F}}} \equiv qL$; and
3. ${\mathcal {F}}$ has isolated singularities and ${\mathcal {F}}$ admits no invariant positive dimensional subvarieties.

Then *L* is ample.

In fact, [13] proves a converse to the above theorem: if *L* is an ample ${\mathbb {Q}}$-Cartier divisor, $n \gg 0$ is sufficiently divisible and ${\mathcal {F}}$ is a general foliation with $K_{{\mathcal {F}}} \equiv nL$, then ${\mathcal {F}}$ admits no invariant positive dimensional subvarieties and has isolated singularities.

Furthermore, we consider the study of singularities of foliations with a non-trivial algebraic part:

### Theorem 1.4

(cf. Theorem 5.4) Let *X* be a ${\mathbb {Q}}$-factorial klt projective variety and let ${\mathcal {F}}$ be a foliation with canonical singularities.

Then the algebraic part of ${\mathcal {F}}$ is induced by an almost holomorphic map.

We expect the Minimal Model Program to have interesting implications for the study of foliation singularities. Indeed, following [11, Theorem 1.6] and [10, Lemma 2.8], we believe that there is a close relation between the classes of singularities of the Minimal Model Program and the dicriticality properties of the foliation. In particular, we expect that canonical singularities satisfy some suitable non-dicritical condition. Theorem 5.4 is a partial confirmation of this in the case of foliations with non-trivial algebraic part (see [12, Conjecture 4.2] in the case of algebraically integrable foliations).

Building off of work of [31] this theorem has implications for the study of foliations where $-K_{{\mathcal {F}}}$ is nef.

### Corollary 1.5

(cf. Corollary 5.5) Let *X* be a smooth projective variety and let ${\mathcal {F}}$ be a foliation with canonical singularities. Suppose that $-K_{\mathcal F}$ is nef and is not numerically trivial.

Then the algebraic part of ${\mathcal {F}}$ is induced by an equidimensional fibration.

## Preliminaries

All our schemes are Noetherian, pure dimensional and separated over an uncountable algebraically closed field *K* of characteristic zero, unless otherwise specified. The results here hold equally well for algebraic spaces and for complex analytic varieties. We will use the fact that many results in the Minimal Model Program for algebraic varieties have been recently generalised to projective morphisms between complex analytic spaces (see [14, 19, 27]).

### Line and divisorial sheaves

Let *X* be a not necessarily reduced $S_2$ scheme. A **line sheaf** *L* on *X* is a coherent rank one $S_2$ sheaf such that there exists a closed subscheme $Z \subset X$ of codimension at least two such that $L\vert _{X {\setminus } Z}$ is locally free.

We recall that if *E* is a coherent $S_2$ sheaf, $Z \subset X$ is a closed subscheme of codimension at least two and $i:X {\setminus } Z \rightarrow X$ is the inclusion then the natural morphism $E \rightarrow i_*E\vert _{X {\setminus } Z}$ is an isomorphism [21, Chapter III, Exercise 3.5]. Moreover, by [35, Proposition 51.8.7] if $Z \subset X$ is a closed subscheme of codimension at least two, $i:X {\setminus } Z \rightarrow X$ is the inclusion and *L* is a locally free sheaf on $X {\setminus } Z$ then $i_*L$ is a coherent sheaf. It is immediate that $i_*L$ is an $S_2$ sheaf. Given an integer *n*, we may define the line sheaf $L^{[n]}$ to be $i_*(L\vert _{X{\setminus } Z}^{\otimes n})$ where $i:X{\setminus } Z \rightarrow X$ is the inclusion.

We denote by $\textrm{LSh}(X)$ the group of isomorphism classes of such sheaves and we define $\textrm{LSh}(X)_{{\mathbb {Q}}} := \textrm{LSh}(X)\otimes {\mathbb {Q}}$ to be the group of ${\mathbb {Q}}$-line sheaves.

If *X* is reduced, a **divisorial sheaf** on *X* is the data of a line sheaf *L* together with a choice of an embedding $L \rightarrow K(X)$ and we denote the group of isomorphism classes of such sheaves by $\textrm{WSh}(X)$. We likewise define the group of $\mathbb {Q}$-divisorial sheaves $\textrm{WSh}(X)_{{\mathbb {Q}}}$.

Consider a scheme *X* and a ${\mathbb {Q}}$-line sheaf *L*. Let $Z \subset X$ be a codimension two subscheme and let $n>0$ be an integer such that $L^{[ n]}\vert _{X{\setminus } Z}$ is locally free on $X {\setminus } Z$. Let *D* be an $S_2$ scheme and let $f:D \rightarrow X$ be a morphism such that $f^{-1}(Z) \subset D$ is of codimension at least two. We define the divisorial pullback $f^wL$ to be $\frac{1}{n}j_*(f^*(L^{[n]}\vert _{X{\setminus } Z}))$ where $j:D {\setminus } f^{-1}(Z) \rightarrow D$ is the inclusion. Note that $f^\omega L$ is a ${\mathbb {Q}}$-line sheaf on *D*. We can likewise define the restriction (and pullback) of a divisorial sheaf. If *L* is the sheaf defined by a ${\mathbb {Q}}$-Cartier divisor, then these notions all agree with the restriction and pullback of ${\mathbb {Q}}$-Cartier divisors. Similarly, if *G* is a prime divisor on *X* such that *mG* is Cartier on $X{\setminus } Z$ for some positive integer *m* and no component of *f*(*D*) is contained in the support of *G*, then we define $f^wG$ to be $\frac{1}{\,}m G'$ where $G'$ is the closure of $f^*(mG|_{X{\setminus } Z})$ in *D*. By linearity, we can extend the definition to any ${\mathbb {Q}}$-divisor on *X* which is ${\mathbb {Q}}$-Cartier on $X{\setminus } Z$.

### Integrable distributions and foliations

Let *X* be a not necessarily reduced $S_2$ scheme. A rank *r* **integrable distribution** ${\mathcal {F}}$ on *X* is the data of a

1. a line sheaf *L*; and
2. a Pfaff field, i.e., a morphism $\phi :\Omega ^r_X \rightarrow L$, satisfying the following integrability condition: in some neighbourhood *U* of the generic point of each irreducible component of *X* there exists a coherent rank *r* sheaf *E* and a surjective morphism $q:\Omega ^1_U \rightarrow E$ such that the *r*-th wedge power of this morphism agrees with $\phi |_U$ and its dual $E^*\hookrightarrow T_U$ is closed under Lie bracket.

We define the **canonical class** of the integrable distribution $${{\mathcal {F}}}$$ to be any Weil divisor $$K_{{\mathcal {F}}}$$ on *X* such that $${\mathcal {O}}_X(K_{{\mathcal {F}}}) \cong L$$. A rank *r* **foliation** on *X* is a rank *r* integrable distribution on *X* whose Pfaff field $$\phi $$ is such that $$\textrm{coker}~ \phi $$ is supported in codimension at least two. Given a rank *r* integrable distribution $${\mathcal {F}}$$ on a normal scheme *X* we define the **singular locus** of $${{\mathcal {F}}}$$, denoted $${{\,\textrm{Sing}\,}}\, {{\mathcal {F}}}$$, to be the co-support of the ideal sheaf defined by the image of the induced map $$(\Omega ^r_X\otimes {\mathcal {O}}_X(-K_{{\mathcal {F}}}))^{**} \rightarrow {\mathcal {O}}_X$$.

For foliations on normal varieties, this definition agrees with the usual definition, see Sect. 2.4 below. Elsewhere in the literature there are differing definitions for foliations on general schemes, we refer to Sect. 3.5.3 for a discussion of this point.

Let *X* be a not necessarily reduced $S_2$ scheme. We define a ${\mathbb {Q}}$**-integrable distribution** of rank *r* on *X* to be the data of a line sheaf *L* on *X* and a non-zero morphism for some $m>0$

$$\begin{aligned} \phi :(\Omega ^r_X)^{\otimes m} \rightarrow L \end{aligned}$$

such that any generic point of *X* admits a neighbourhood *U* and an integrable distribution ${\mathcal {F}}$ on *U* defined by the Pfaff field $\phi _0:\Omega ^r_U \rightarrow {\mathcal {O}}_U(K_{\mathcal F})$ and such that $\phi |_U = \phi _0^{\otimes m}$. We say that *m* is the **index** of the ${\mathbb {Q}}$-integrable distribution. We will refer to ${\mathcal {F}}$ as the **associated integrable distribution**.

We make note of the following:

#### Lemma 2.1

Let *X* be an $S_2$ scheme, let *E* be a coherent sheaf on *X* and let $L_1, L_2$ be line sheaves on *X* with morphisms $\psi _i:E \rightarrow L_i$ such that

1. $\psi _1 = \psi _2$ at the generic points of *X*; and
2. $\textrm{coker}~ \psi _1$ is supported in codimension at least two.

Then there exists a non-zero morphism $L_1 \rightarrow L_2$. In particular, if *X* is normal then there exists a uniquely defined effective Weil divisor *B* such that $L_2 = L_1\otimes \mathcal O_X(B)$.

#### Proof

We may freely remove subschemes of codimension at least two from *X* and so we may assume that $L_1$ and $L_2$ are locally free and that $\psi _1$ is surjective. Let *Q* be the kernel of $\psi _1$. By item (1) we have that $\psi _2(Q) \subset L_2$ is a torsion subsheaf, and is therefore identically zero. Our result then follows. $\square$

#### Lemma 2.2

Let *X* be an $S_2$ scheme, let $\mathcal F^{\circ }$ be an integrable distribution on *X* and let $U \subset X$ be a dense open subset.

1. If $X {\setminus } U$ is of codimension at least two then ${\mathcal {F}}^{\circ }$ is uniquely determined by its restriction to *U*.
2. If ${\mathcal {F}}^{\circ }$ is a foliation, then it is uniquely determined by its restriction to *U*.
3. Suppose that *X* is normal and that ${\mathcal {G}}_U$ is a foliation on *U*. Then there exists a unique foliation ${\mathcal {G}}$ on *X* whose restriction to *U* is ${\mathcal {G}}_U$.
4. Suppose that *X* is normal. Then there exists a unique foliation ${\mathcal {F}}$ on *X* which agrees with ${\mathcal {F}}^\circ$ at the generic point of *X*. In particular, there exists a canonically defined effective divisor *B* such that $K_{{\mathcal {F}}}+B \sim K_{{\mathcal {F}}^\circ }$.

#### Proof

All the items are easy consequences of Lemma 2.1. $\square$

In Item (4), we will refer to ${{\mathcal {F}}}$ as the foliation induced by the integrable distribution ${\mathcal {F}}^\circ$.

### Invariant subschemes

Given an $S_2$ scheme *X* and a rank *r* integrable distribution ${\mathcal {F}}$ on *X*, we say that an irreducible subscheme $W \subset X$ is ${\mathcal {F}}$**-invariant** (or simply **invariant** if the integrable distribution is understood) if $K_{{\mathcal {F}}}$ is Cartier at the generic point of *W* and in a neighbourhood of the generic point of *W* there is a factorisation 
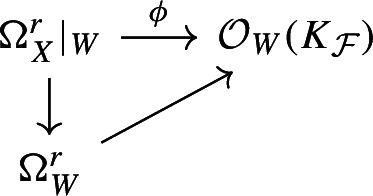
 More generally, we say that a subscheme $W \subset X$ is $\mathcal {F}$-invariant if each irreducible component is invariant.

Given an $S_2$ scheme *X*, an integrable distribution ${\mathcal {F}}$ and a divisor *D* on *X*, such that either *D* is $\mathcal {F}$-invariant or every component of *D* is not ${{\mathcal {F}}}$-invariant, we define $\epsilon ({\mathcal {F}}, D) {:=} 0$ if *D* is $\mathcal F$-invariant, and $\epsilon ({\mathcal {F}}, D) {:=} 1$ if each component of *D* is not ${{\mathcal {F}}}$-invariant. When ${\mathcal {F}}$ is clear from context we will write $\epsilon (D)$ in place of $\epsilon ({\mathcal {F}}, D)$.

Given any ${\mathbb {Q}}$-divisor *D* on *X*, we denote $D_{\textrm{inv}}$ to be the part of *D* supported on invariant divisors and $D_{\mathrm{n-inv}} {:=} D-D_{\textrm{inv}}$.

#### Remark 2.3

The definition above should be compared with [3, Definition 3.4]. Away from ${{\,\textrm{Sing}\,}}X \cup {{\,\textrm{Sing}\,}}{\mathcal {F}}$ these two definitions agree.

### Foliations on normal varieties

When *X* is a normal scheme, the above definition of foliation is equivalent to the usual definition of a foliation ${\mathcal {F}}$ in terms of a saturated subsheaf $T_{{\mathcal {F}}} \subset T_X$ closed under Lie bracket. In this case, we may take $U{:=} X{\setminus } ({{\,\textrm{Sing}\,}}X\cup {{\,\textrm{Sing}\,}}\, {{\mathcal {F}}})$, $E{:=} T_{{{\mathcal {F}}}}^*|_U$ and $L {:=} {\mathcal {O}}_X(K_{{\mathcal {F}}})$. To verify this equivalence we first note that by Lemma 2.2 it suffices to verify this equivalence away from a subvariety of codimension at least two and, in particular, we may replace *X* by *U*. Given the saturated subsheaf $T_{\mathcal F} \subset T_X$ we get a Pfaff field by considering the morphism

$$\begin{aligned} \Omega ^r_X \rightarrow \left( \bigwedge ^r T_{{\mathcal {F}}}\right) ^{*} \cong {\mathcal {O}}_X(K_{{\mathcal {F}}}) \end{aligned}$$

where *r* is the rank of ${\mathcal {F}}$.

Conversely, consider a Pfaff field $\phi :\Omega ^r_X \rightarrow L$ satisfying our integrability condition. By assumption, over a dense open subset *U* of *X*, we have a surjective morphism $\Omega ^1_U \rightarrow E$ whose dual defines a foliation on *U*, which extends uniquely to a foliation on *X*.

#### Lemma 2.4

Let *X* be a normal scheme, let ${\mathcal {F}}$ be a rank *r* foliation on *X* and let $W \subset X$ be an irreducible subscheme such that $T_{{\mathcal {F}}}$ is locally free in a neighbourhood of the generic point of *W*. Let $I_W$ be the ideal of *W*.

If in a neighbourhood of the generic point of *W* we have $\partial (I_W) \subset I_W$ for all local sections $\partial \in T_{{\mathcal {F}}}$ then *W* is ${{\mathcal {F}}}$-invariant.

#### Proof

Everything is local about the generic point of *W*, so we may freely replace *X* by a neighbourhood of the generic point of *W* and therefore we may assume that $T_{{\mathcal {F}}}$ is locally free. Let $\partial _1, \dots , \partial _r$ be generators of $T_{{\mathcal {F}}}$.

Suppose that $\partial _i(I_W) \subset I_W$ for all *i* then $(\partial _1 \wedge \dots \wedge \partial _r)(df \wedge \beta )$ vanishes along *W* for any $f \in I_W$ and any $(r-1)$-form $\beta$. In particular, $(dI_W\wedge \Omega _X^{r-1})\vert _W = \ker (\Omega ^r_X\vert _W \rightarrow \Omega ^r_W)$ is contained in the kernel of $\Omega ^r_X\vert _W \rightarrow {\mathcal {O}}_W(K_{\mathcal F})$. This implies that in a neighbourhood of the generic point of W, the morphism factors through $\Omega ^r_X\vert _W \rightarrow \Omega ^r_W$ and so *W* is ${{\mathcal {F}}}$-invariant. $\square$

When *X* is smooth the above Lemma is proven in [2, Lemma 2.7]. Moreover it is shown there that if *W* is $\mathcal {F}$-invariant and not contained in the singular locus of ${\mathcal {F}}$ then in a neighbourhood of the generic point of *W* we have $\partial (I_W) \subset I_W$ for all local sections $\partial \in T_{{\mathcal {F}}}$.

### Singularities of foliations from the perspective of the MMP

We refer to [25] for general notions of singularities coming from the MMP. We refer to [11, §2.4] for a recollection on the definition of foliation singularities from the perspective of the MMP. We say that a variety *X* is **potentially klt** if there exists a ${\mathbb {Q}}$-divisor $\Gamma \ge 0$ such that $(X, \Gamma )$ is klt.

If *D* is a ${\mathbb {Q}}$-divisor on a normal variety *X* and $\Sigma$ is a prime divisor in *X*, then we denote by $m_\Sigma D$ the coefficient of *D* along $\Sigma$.

#### Lemma 2.5

Let *X* be a normal variety, let ${\mathcal {F}}$ be a foliation on *X* and let $\Delta \ge 0$ be a ${\mathbb {Q}}$-divisor on *X* such that $({\mathcal {F}}, \Delta )$ is log canonical.

Then no component of $\Delta$ is ${\mathcal {F}}$-invariant.

#### Proof

It follows immediately from [11, Remark 2.3]. $\square$

### Algebraically integrable foliations

A dominant map $\sigma :X\dashrightarrow Y$ between normal varieties is called **almost holomorphic** if there exist dense Zariski open subsets $U\subset X$ and $V\subset Y$ such that the induced map $\sigma |_U:U\rightarrow V$ is a proper morphism.

Let $\sigma :Y\dashrightarrow X$ be a dominant map between normal varieties and let ${\mathcal {F}}$ be a foliation of rank *r* on *X*. We denote by $\sigma ^{-1}{\mathcal {F}}$ the **induced foliation** on *Y* (e.g. see [18, Section 3.2]). If $T_{{\mathcal {F}}}=0$, i.e. if ${\mathcal {F}}$ is the foliation by points on *X*, then we refer to $\sigma ^{-1}{\mathcal {F}}$ as the **foliation induced by** $\sigma$ and we denote it by $T_{X/Y}$. In this case, the foliation $\sigma ^{-1}{\mathcal {F}}$ is called **algebraically integrable**.

Let $f:X\rightarrow Z$ be a morphism between normal varieties and let ${\mathcal {F}}$ be the induced foliation on *X*. If *f* is equidimensional, then we define the **ramification divisor** *R*(*f*) of *f* as

$$\begin{aligned} R(f)=\sum _D (f^*D-f^{-1}(D)) \end{aligned}$$

where the sum runs through all the prime divisors of *Z*. Note that, since *f* is equidimensional, the pullback $f^*D$ is well defined even though *D* might not be ${\mathbb {Q}}$-Cartier. In this case, we have

$$\begin{aligned} K_{{\mathcal {F}}} \sim K_{X/Z}-R(f) \end{aligned}$$

(e.g. see [16, Notation 2.7 and §2.9]).

Let *X* be a normal variety and let ${{\mathcal {F}}}$ be a foliation on *X*. Then ${\mathcal {F}}$ is called **purely transcendental** if there is no positive dimensional algebraic subvariety passing through the general point of *X*, which is tangent to ${{\mathcal {F}}}$. In general, by [16, Definition 2.3] (see also [4, Definition 2]) it follows that for any foliation ${{\mathcal {F}}}$ on *X* there exists a dominant map $\sigma :X\dashrightarrow Y$ and a purely transcendental foliation ${{\mathcal {G}}}$ on *Y* such that $\mathcal {F}=\sigma ^{-1}{{\mathcal {G}}}$. Note that *Y* and ${{\mathcal {G}}}$ are unique up to birational equivalence. The foliation ${{\mathcal {H}}}$ induced by $\sigma$ is called the **algebraic part of** ${{\mathcal {F}}}$.

## Adjunction

### Lifting derivations on the normalisation

The goal of this subsection is to prove the following:

#### Proposition 3.1

Let *X* be a reduced scheme and let $n:{\tilde{X}} \rightarrow X$ be its normalisation. Suppose that *L* is a locally free sheaf of rank one on *X* and that we have a morphism $\phi :(\Omega ^r_X)^{\otimes m} \rightarrow L$ for some $r, m \ge 0$.

Then there is a natural morphism ${\tilde{\phi }} :(\Omega ^r_{{\tilde{X}}})^{\otimes m} \rightarrow n^*L$ which agrees with $\phi$ at any generic point of ${\tilde{X}}$.

Using the same notation as in the Proposition, we will call ${\tilde{\phi }}$ the **lift ** of $\phi$.

We follow closely the proofs of [23, Theorem 2.1.1] and [5, Proposition 4.5]. Given an integral domain *A* we denote by *K*(*A*) the field of fractions of *A*. Let *M* be an *A*-module and let $r,m\ge 0$. Then an *A*-linear map

$$\begin{aligned} \phi :(\Omega ^r_{A})^{\otimes m} \rightarrow M \end{aligned}$$

induces a map

$$\begin{aligned} \partial :K(A)^{\oplus rm}\rightarrow M\otimes _{A}K(A)\quad \text {such that}\quad \partial (A^{\oplus rm})\subset M. \end{aligned}$$

Note that $\partial$ is a derivation if $r=m=1$.

We begin with the following two Lemma:

#### Lemma 3.2

Let *A* be a Noetherian integral *K*-algebra, let $B \subset A$ be a Noetherian subalgebra and let $\partial :B \rightarrow A$ be a derivation. Let $A'$ (resp. $B'$) be the integral closure of *A* in *K*(*A*) (resp. of *B* in *K*(*B*)).

Then $\partial$ lifts to a derivation $\partial ':B' \rightarrow A'$.

#### Proof

The proof of [32, Theorem, §3] works equally well here. Indeed, as noted in [32, Footnote 2], the only thing that is needed in the proof is that the differential operator $E{:=} e^{t\partial }$ defines an injective map $E:K(B)[[t]] \rightarrow K(A)[[t]]$, which is immediate since *B* is a subalgebra of *A*. $\square$

#### Lemma 3.3

Let *X* be a normal scheme and let *E* be a coherent sheaf on *X*. Let *m* be a positive integer and let $s:E^{\otimes m} \rightarrow {\mathcal {O}}_X$ be a morphism. Assume that there exists a morphism $t:E\rightarrow \mathcal O_{X}$ and a rational function $\rho \in K(X)$ such that $s = \rho t^{\otimes m}$.

Then there exists a cyclic Galois cover $\sigma :{\overline{X}} \rightarrow X$ and a morphism ${\bar{t}}:\sigma ^*E \rightarrow {\mathcal {O}}_{{\overline{X}}}$ such that ${\bar{t}}^{\otimes m} = \sigma ^*s$.

#### Proof

Let ${\overline{X}}$ to be the normalisation of *X* in $K(X)(\root m \of {\rho })$ and let $\sigma :{\overline{X}}\rightarrow X$ be the induced morphism. Then there exists a rational function *z* on ${\overline{X}}$ such that if ${\bar{t}}=z \sigma ^*t$ then ${\bar{t}}:\sigma ^*E \rightarrow {\mathcal {O}}_{{\overline{X}}}$ is a morphism such that ${\bar{t}}^{\otimes m} = \sigma ^*s$. $\square$

We call the morphism ${\bar{t}}$ constructed in Lemma 3.3 the *m***-th root** of *s*.

#### Proof of Propostion 3.1

The claim is local on *X* so we may freely assume that $X = \text {Spec}~A$ is affine and $L \cong {\mathcal {O}}_X$. Moreover, it suffices to prove the existence of the lift after restricting to an irreducible component of *X* and so we may freely assume that *X* is integral. Let $A'$ be the integral closure of *A* in *K*(*A*) and define $X' {:=} \text {Spec}\, {A'}$ and let $n:X' \rightarrow X$ be the normalisation morphism. Using the same argument as in the proofs of [5, Lemma 4.3 and Proposition 4.5], we may assume that *A* and $A'$ are complete one dimensional local rings.

Let us first suppose that $r= 1$. The map $\phi :(\Omega ^1_X)^{\otimes m}\rightarrow {\mathcal {O}}_X$ induces a map $\phi ':(n^*\Omega ^1_X)^{\otimes m} \rightarrow {\mathcal {O}}_{X'}$. Let $t:n^*\Omega ^1_X \rightarrow {\mathcal {O}}_{X'}$ be a non-zero map and let $\rho \in K(X')$ be a rational function such that $\phi '=\rho t^{\otimes m}$. Then Lemma 3.3 implies the existence of a cover $\sigma :{\overline{X}} = \text {Spec}\, {{\overline{A}}} \rightarrow X'$ associated to $\phi '$. Let $\psi :\sigma ^*n^*\Omega ^1_X \rightarrow {\mathcal {O}}_{{\overline{X}}}$ be the *m*-th root of $\phi$.

Observe that $\psi$ corresponds to a derivation $\partial _{\psi }:A \rightarrow {\overline{A}}$. By Lemma 3.2 this lifts to a derivation $\partial '_{\psi }:A' \rightarrow {\overline{A}}$. This in turn implies that $\psi$ lifts to a morphism $\rho :\sigma ^*\Omega ^1_{X'} \rightarrow {\mathcal {O}}_{{\overline{X}}}$. Finally note that $\rho ^{\otimes m}$ is *G*-invariant and so descends to a morphism $(\Omega ^1_{X'})^{\otimes m} \rightarrow {\mathcal {O}}_{X'}$, which is precisely our required lifting of $\phi$.

To prove the claim, by following the proof of [5, Proposition 4.5], we may proceed by induction on *r* and reduce the proof of the statement for general $r \ge 1$ to the case $r = 1$, proven above. $\square$

#### Lemma 3.4

Let *X* be an integral scheme and let $\phi :\Omega _X^r \rightarrow L$ be a Pfaff field where *L* is a locally free sheaf of rank one on *X*. Let $n:X' \rightarrow X$ be the normalisation. Let $P \subset X$ be a codimension one subscheme and assume that $\phi$ vanishes along $P \subset X$.

Then $\phi ':\Omega ^r_{X'} \rightarrow n^*L$ vanishes along $n^{-1}(P)$ where $\phi '$ is the lift of $\phi$ to the normalisation.

#### Proof

The claim is local about the generic point of *P*, so up to shrinking *X* we may freely assume that $X = \text {Spec}\, A$ is affine and $X' = \text {Spec}\, A'$. We may argue as in the proof of [5, Proposition 4.5] to reduce to the case where *A* and $A'$ are complete one dimensional local rings with coefficients fields *K* and $K'$, respectively. We now proceed by induction on *r*. When $r = 1$ the claim follows from [15, Lemme 1.2] (note that the cited Lemma is stated for varieties, but applies equally well to reduced schemes). So suppose that $r \ge 2$. Let *t* be a uniformising parameter of $A'$. To prove our claim, it suffices to show that $\phi '(dx_1\wedge \dots \wedge dx_r)=0$ for $x_1,\dots ,x_r\in K'\cup \{t\}$. Since $dt\wedge \dots \wedge dt = 0$ we may assume (up to relabeling) that $x_1 \in K'$. Since $K \subset K'$ is a finite extension we may find $Q(s) = \sum _{i = 0}^c a_is^i \in K[s]$ such that $Q(x_1) =0$ and $Q'(x_1) \ne 0$. On one hand we have that

$$\begin{aligned} 0 =dQ(x_1)\wedge dx_2 \wedge \dots \wedge dx_r= &   Q'(x_1)dx_1\wedge \dots \wedge dx_r\\    &   + \sum _{i = 0}^c x_1^ida_i \wedge dx_2 \wedge \dots \wedge dx_r \end{aligned}$$

and so

$$\begin{aligned} \phi '(dx_1\wedge \dots \wedge dx_r) = -\frac{1}{Q'(x_1)}\phi ' \left( \sum _{i = 0}^c x_1^ida_i \wedge dx_2 \wedge \dots \wedge dx_r \right) . \end{aligned}$$

On the other hand, $\phi (da_i \wedge \cdot ):\Omega ^{r-1}_X \rightarrow L$ defines a Pfaff field of rank $r-1$ and so our induction hypothesis implies that

$$\begin{aligned} \phi ' \left( \sum _{i = 0}^c x_1^ida_i \wedge dx_1 \wedge \dots \wedge dx_r \right) \end{aligned}$$

vanishes along $n^{-1}(P)$ and we may conclude. $\square$

### Construction of the different

#### Lemma 3.5

Let *X* be a normal scheme, let ${\mathcal {F}}$ be a foliation of rank *r* on *X*, let $\iota :D \hookrightarrow X$ be a reduced subscheme of codimension one and suppose that either every component of *D* is ${{\mathcal {F}}}$-invariant or that every component of *D* is not ${{\mathcal {F}}}$-invariant. Let $n:S \rightarrow D$ be the normalisation and suppose there exist

1. a subscheme *Z* of *X* such that $Z \cap D$ is of codimension at least two in *D*; and
2. a ${\mathbb {Q}}$-divisor $\Delta \ge 0$ on *X* which does not contain any component of *D* in its support and such that for some sufficiently divisible positive integer *m* we have that $m(K_{{\mathcal {F}}}+\Delta )$ and $\epsilon (D)D$ are Cartier on $X {\setminus } Z$.

Then, there exists a canonically defined ${\mathbb {Q}}$-integrable distribution of rank $r-\epsilon (D)$ and index *m* on *S*, given by a morphism

$$\begin{aligned} \psi _S:(\Omega ^{r-\epsilon (D)}_S)^{\otimes m} \rightarrow n^w{\mathcal {O}}_X(m(K_{{\mathcal {F}}}+\Delta +\epsilon (D)D)). \end{aligned}$$

#### Proof

It suffices to prove the Lemma away from a subvariety of codimension at least two in *D*, and so we may freely assume that for some sufficiently divisible positive integer *m*, we have that $m(K_{{\mathcal {F}}}+\Delta )$ and $\epsilon (D)D$ are Cartier.

Taking the *m*-th tensor power of the Pfaff field defining $\mathcal F$ and composing with the inclusion ${\mathcal {O}}_X(mK_{{\mathcal {F}}}) \rightarrow {\mathcal {O}}_X(m(K_{{\mathcal {F}}}+\Delta ))$ we get a morphism $\phi :(\Omega ^r_X)^{\otimes m} \rightarrow \mathcal O_X(m(K_{{\mathcal {F}}}+\Delta ))$.

Suppose first that *D* is ${{\mathcal {F}}}$-invariant. Let $N {:=} \textrm{ker}~ ((\Omega ^r_X)^{\otimes m}\vert _D \rightarrow (\Omega ^r_D)^{\otimes m})$ and let $\phi \vert _D$ be the restriction of $\phi$ to *D*. By definition $\phi \vert _D(N)$ vanishes at the generic point of *D*, and since $m(K_{{\mathcal {F}}}+\Delta )$ is Cartier and *D* is reduced, it follows that $\phi \vert _D(N) = 0$. Therefore we have a morphism $\psi :(\Omega ^r_D)^{\otimes m} \rightarrow {\mathcal {O}}_D(m(K_{{\mathcal {F}}}+\Delta ))$.

By Lemma  2.4, we have a commutative diagram in a neighbourhood *U* of the generic point of *D*
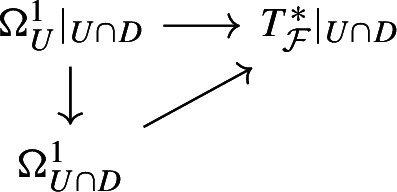
 and the integrability condition follows immediately.

Now suppose that *D* is not ${{\mathcal {F}}}$-invariant. We define a morphism

$$\begin{aligned} \psi ':(\Omega _D^{r-1})^{\otimes m} \rightarrow \mathcal O_D(m(K_{{\mathcal {F}}}+\Delta +D)) \end{aligned}$$

by

$$\begin{aligned} \psi '(\alpha _1\otimes \dots \otimes \alpha _m) := \frac{\phi (df \wedge {\tilde{\alpha }}_1 \otimes \dots \otimes df \wedge {\tilde{\alpha }}_m)}{f^m}\vert _D \end{aligned}$$

for any local sections $\alpha _1, \dots , \alpha _m$ of $\Omega ^{r-1}_D$, where *f* is a local equation of *D*, ${\tilde{\alpha }}_i$ is any local lift of $\alpha _i$ to $\Omega ^{r-1}_X$, and $\frac{\phi (df \wedge {\tilde{\alpha }}_1 \otimes \dots \otimes df \wedge {\tilde{\alpha }}_m)}{f^m}$ is considered as a section of ${\mathcal {O}}_X(m(K_{\mathcal F}+\Delta +D))$. We claim that this morphism is independent of our choice of *f*. Indeed, if $f'$ is another local equation of *D* then $f' = uf$ where *u* is a unit. We compute that

$$\begin{aligned} \frac{\phi (df'\wedge {\tilde{\alpha }}_1 \otimes \dots \otimes df'\wedge {\tilde{\alpha }}_m)}{(f')^m} = \frac{\phi (df\wedge {\tilde{\alpha }}_1 \otimes \dots \otimes df \wedge {\tilde{\alpha }}_m)}{f^m}+ \frac{\phi (\omega _0)}{f^{m-1}}, \end{aligned}$$

where $\omega _0$ is a local section of $\Omega ^r_X$. Observe that $\frac{\phi (\omega _0)}{f^{m-1}}$ is a section of $\mathcal O_X(m(K_{{\mathcal {F}}}+\Delta )+(m-1)D)$, and so it vanishes along *D* when considered as a section of ${\mathcal {O}}_X(m(K_{\mathcal F}+\Delta +D))$. Thus,

$$\begin{aligned} \frac{\phi (df'\wedge {\tilde{\alpha }}_1 \otimes \dots \otimes df'\wedge {\tilde{\alpha }}_m)}{(f')^m}|_D = \frac{\phi (df\wedge {\tilde{\alpha }}_1 \otimes \dots \otimes df \wedge {\tilde{\alpha }}_m)}{f^m}|_D, \end{aligned}$$

as required. Likewise, it is easy to check that $\psi '$ is independent of the choice of $\tilde{\alpha _i}$.

Since *D* is not ${{\mathcal {F}}}$-invariant, by Lemma 2.4 the composition

$$\begin{aligned} {{\mathcal {O}}}_{U\cap D}(-D)\rightarrow \Omega ^1_U|_{U\cap D}\rightarrow T_{\mathcal {F}}^*|_{U\cap D} \end{aligned}$$

is non-zero. Let *E* be its cokernel. Then, the induced map

$$\begin{aligned} \Omega ^1_{U\cap D}\rightarrow E \end{aligned}$$

satisfies the integrability condition.

In either case, by Proposition 3.1 we have a lift of $\psi$ or $\psi '$ to

$$\begin{aligned}{\tilde{\psi }}:(\Omega ^{r-\epsilon (D)}_S)^{\otimes m} \rightarrow n^*{\mathcal {O}}_X(m(K_{{\mathcal {F}}}+\Delta +\epsilon (D)D)) \end{aligned}$$

which necessarily satisfies our integrability condition and we may conclude. $\square$

#### Remark 3.6

Set-up as in Lemma 3.5. If in addition we have that *D* is $S_2$, then using the same proof as in the Lemma, it follows that there exists a canonically defined ${\mathbb {Q}}$-integrable distribution of rank $r-\epsilon (D)$ and index *m* on *D*, given by a morphism

$$\begin{aligned} \psi _D:(\Omega _D^{r-\epsilon (D)})^{\otimes m} \rightarrow \iota ^w\mathcal O_X(m(K_{{\mathcal {F}}}+\Delta +\epsilon (D)D)). \end{aligned}$$

#### Proposition-Definition 3.7

Let *X* be a normal scheme, let ${\mathcal {F}}$ be a foliation of rank *r* on *X*, and let $\iota :D \hookrightarrow X$ be an integral subscheme of codimension one. Let $n:S \rightarrow D$ be the normalisation. Suppose that there exist a subscheme *Z* of *X* such that $Z \cap D$ is of codimension at least two in *D* and a ${\mathbb {Q}}$-divisor $\Delta \ge 0$ on *X* which does not contain *D* in its support and such that $K_{{\mathcal {F}}}+\Delta$ and $\epsilon (D)D$ are ${\mathbb {Q}}$-Cartier on $X {\setminus } Z$.

Then, there exists a canonically defined **restricted foliation** ${\mathcal {F}}_{S}$ on *S*, and a canonically defined $\mathbb {Q}$-divisor $\textrm{Diff}_S({\mathcal {F}}, \Delta ) \ge 0$, called the **different**, such that

$$\begin{aligned} n^w(K_{{\mathcal {F}}}+\Delta +\epsilon (D)D) \sim _{{\mathbb {Q}}} K_{\mathcal F_S}+\textrm{Diff}_S({\mathcal {F}}, \Delta ). \end{aligned}$$

If $\Delta =0$ then we denote $\textrm{Diff}_S({{\mathcal {F}}},0)$ simply as $\textrm{Diff}_S({{\mathcal {F}}})$.

#### Proof

It suffices prove the Proposition away from a subvariety of codimension at least two in *D*, and so we may freely assume that $K_{{\mathcal {F}}}+\Delta$ and $\epsilon (D)D$ are ${\mathbb {Q}}$-Cartier.

We first construct the restricted foliation on *S*. By Lemma 2.2 it suffices to define the restricted foliation ${\mathcal {F}}_S$ at the generic point of *S*. We may therefore assume that $K_{{\mathcal {F}}}$ and *D* are both Cartier and therefore may apply Lemma 3.5 to produce ${\mathcal {F}}_S$.

Let *m* be a sufficiently divisible positive integer *m* such that $m(K_{{\mathcal {F}}}+\Delta )$ and $m\epsilon (D)D$ are Cartier. Then, by Lemma 3.5, we have a morphism at the generic point $\eta$ of *S*,

$$\begin{aligned} \psi _\eta :(\Omega ^{r-\epsilon (D)}_S)^{\otimes m}\vert _\eta \rightarrow {\mathcal {O}}_S(m(K_{{\mathcal {F}}}+\Delta +\epsilon (D)D))\vert _\eta \end{aligned}$$

which agrees with $(\Omega ^{r-\epsilon (D)}_S)|_{\eta }^{\otimes m} \rightarrow {\mathcal {O}}_\eta (mK_{{\mathcal {F}}_S})$. If we can show that, after possibly replacing *m* by a larger multiple, $\psi _{\eta }$ extends to a morphism on all of *S*, call it $\psi _S$, then by Lemma 2.1 there exists an effective Weil divisor *B* such that

$$\begin{aligned} mK_{{{\mathcal {F}}}_S}+B\sim m(K_{{{\mathcal {F}}}}+\Delta +\epsilon (D)D)|_S. \end{aligned}$$

Thus, it is enough to define $\textrm{Diff}_S({{\mathcal {F}}},\Delta ){:=} \frac{1}{\,}m B$.

Note that if *D* is ${{\mathcal {F}}}$-invariant, then the existence of $\psi _S$ is guaranteed by Lemma  3.5. Thus, we may assume that *D* is not ${{\mathcal {F}}}$-invariant. To check that $\psi _{\eta }$ extends as a morphism, it suffices to do so locally. Let $P\in D$ be a closed point. Then there exists a quasi-étale cyclic cover $q:V\rightarrow U$ with Galois group *G*, where *U* is a neighbourhood of *P* in *X*, and such that $q^*D$ is a Cartier divisor. Let *m* be the order of *G*. After possibly replacing *q* by a higher cover, we may assume that *m* does not depend on $P\in D$. Let ${{\mathcal {F}}}_V:= q^{-1}{{\mathcal {F}}}|_U$ be the foliation induced on *V*, let $D_V:= q^{-1}(D \cap U)$ and let $\Delta _V$ be a ${\mathbb {Q}}$-divisor on *V* such that $K_{\mathcal {F}_V}+\Delta _V=q^*(K_{{{\mathcal {F}}}}+\Delta )$. By assumption, we may assume that *q* is étale in a neighborhood of any general point of $U\cap \textrm{Supp}\, \Delta$. Thus, $\Delta _V\ge 0$. Let $\nu :S_V\rightarrow D_V$ be the normalisation of $D_V$ and let $p:S_V\rightarrow S_U:=n^{-1}(U\cap D)$ be the induced morphism. Note that $D_V$ is not ${{\mathcal {F}}}_V$-invariant.

Since $m(K_{{{\mathcal {F}}}_V}+\Delta _V)$ and $D_V$ are Cartier on *V*, by Lemma 3.5 there exists a ${\mathbb {Q}}$-integrable distribution of rank $r-1$ and index *m* on $S_V$, given by a morphism

$$\begin{aligned} \psi _{S_V}:(\Omega _{S_V}^{r-1})^{\otimes m}\rightarrow \nu ^*\mathcal O_{D_V}(m(q^*(K_{{{\mathcal {F}}}}+\Delta +D))). \end{aligned}$$

Since $S_U$ is $S_2$, and $m(K_{{{\mathcal {F}}}}+\Delta +D)$ is Cartier on $S_U$ the map $\psi _\eta |_{S_U}$ extends to $S_U$ if and only if the map $p^*(\psi _\eta |_{S_U})$ extends to $S_V$. If $\sigma :p^*(\Omega ^{r-1}_{S_U})^{\otimes m}\rightarrow (\Omega _{S_V}^{r-1})^{\otimes m}$ denotes the natural map then the composition

$$\begin{aligned} \psi _{S_V}\circ \sigma :p^*(\Omega ^{r-1}_{S_U})^{\otimes m}\rightarrow \nu ^*{\mathcal {O}}_{D_V}(m(q^*(K_{{{\mathcal {F}}}}+\Delta +D))) \end{aligned}$$

is an extension of $p^*(\psi _\eta |_{S_U})$. Thus, our claim follows. $\square$

#### Remark 3.8

Set-up as in Proposition-Definition 3.7. If in addition we have that *D* is $S_2$ then there exists a $\mathbb {Q}$-integrable distribution of rank $r-\epsilon (D)$ and index *m* on *D*, given by the morphism

$$\begin{aligned} \psi _D:(\Omega ^{r-\epsilon (D)}_D)^{\otimes m} \rightarrow \iota ^w{\mathcal {O}}_X(m(K_{{\mathcal {F}}}+\Delta +\epsilon (D)D)) \end{aligned}$$

and whose associated integrable distribution on *D* coincides with the restricted foliation on *S* at any generic point of *D*. Indeed, using the same construction as in Proposition-Definition 3.7, by Lemma 3.5 and Remark 3.6 we may assume that *D* is not ${{\mathcal {F}}}$-invariant and that we have a morphism

$$\begin{aligned} (\Omega ^{r-\epsilon (D)}_{D_V})^{\otimes m} \rightarrow \mathcal O_{D_V}(q^*(m(K_{{{\mathcal {F}}}}+\Delta +D))). \end{aligned}$$

Proceeding as in the proof of Proposition-Definition 3.7, it follows that there exists a morphism

$$\begin{aligned} (\Omega ^{r-1}_{D_U})^{\otimes m} \rightarrow \iota ^w(\mathcal O_U(m(K_{{\mathcal {F}}}+\Delta +D))) \end{aligned}$$

and so we may conclude.

#### Remark 3.9

Set-up as in Proposition-Definition 3.7. If in addition we have that $K_{{\mathcal {F}}}+\Delta$ and $\epsilon (D)D$ are ${\mathbb {Q}}$-Cartier then

$$\begin{aligned} n^*(K_{{\mathcal {F}}}+\Delta +\epsilon (D)D) \sim _{{\mathbb {Q}}} K_{{\mathcal {F}}_S}+\textrm{Diff}_S({\mathcal {F}}, \Delta ). \end{aligned}$$

#### Remark 3.10

Set up as in Definition-Proposition 3.7. Let $B\ge 0$ be a ${\mathbb {Q}}$-divisor which does not contain *D* in its support and such that *B* is ${\mathbb {Q}}$-Cartier on $X {\setminus } Z$. Then, from the construction it follows immediately that

$$\begin{aligned} \textrm{Diff}_S({{\mathcal {F}}},\Delta +B) = \textrm{Diff}_S({{\mathcal {F}}},\Delta )+n^wB. \end{aligned}$$

#### Remark 3.11

Set up as in Definition-Proposition 3.7. We can compute the different using resolutions. Indeed, suppose that $\pi :X' \rightarrow X$ is a log resolution of $(X, D+\textrm{Supp}\, \Delta )$. Note that $\pi$ is not necessarily a reduction of singularities of ${\mathcal {F}}$. We may write

$$\begin{aligned} K_{{\mathcal {F}}'}+\Delta '+ \epsilon (D)D'+E = \pi ^*(K_{\mathcal F}+\Delta +\epsilon (D)D) \end{aligned}$$

where *E* is $\pi$-exceptional, ${\mathcal {F}}' {:=} \pi ^{-1}{\mathcal {F}}$, $D'= \pi _*^{-1}D$ and $\Delta ' = \pi _*^{-1}\Delta$. Let *S* be the normalisation of *D* and let $\mu :D' \rightarrow S$ be the induced morphism.

Then

$$\begin{aligned} \mu _*(\textrm{Diff}_{D'}({{\mathcal {F}}}',\Delta ')+E|_{D'}) = \textrm{Diff}_S(\mathcal {F},\Delta ). \end{aligned}$$

In the case of higher codimension invariant centres, we also have a sub-adjunction statement:

#### Proposition-Definition 3.12

Let *X* be a normal complex analytic space, let ${\mathcal {F}}$ be a foliation of rank *r* on *X*, and let $\iota :D \rightarrow X$ be a closed analytic subset. Let $n:S \rightarrow D$ be the normalisation.

Suppose that

1. there exists a ${\mathbb {Q}}$-divisor $\Delta \ge 0$ such that $K_{{\mathcal {F}}}+\Delta$ is ${\mathbb {Q}}$-Cartier;
2. *D* is not contained in $\textrm{Supp }\,\Delta \cup {{\,\textrm{Sing}\,}}\, {{\mathcal {F}}}$;
3. $T_{{\mathcal {F}}}$ is locally free in a neighbourhood of the general point of *D*; and
4. *D* is $${\mathcal {F}}$$-invariant.

Then there exists a canonically defined **restricted foliation** ${\mathcal {F}}_{S}$ on *S*, and a canonically defined $\mathbb {Q}$-divisor $\textrm{Diff}_S({{\mathcal {F}}},\Delta ) \ge 0$, called the **different**, such that

$$\begin{aligned} n^*(K_{{\mathcal {F}}}+\Delta ) \sim _{{\mathbb {Q}}} K_{{\mathcal {F}}_S}+\textrm{Diff}_S({{\mathcal {F}}},\Delta ). \end{aligned}$$

#### Proof

We first construct the restricted foliation ${\mathcal {F}}_S$. Set

$$\begin{aligned} N {:=} \ker (\Omega ^1_X \rightarrow T_{{\mathcal {F}}}^*) \end{aligned}$$

and let $N_S$ be the image of $n^*N$ under the natural map $n^*\Omega ^1_X \rightarrow \Omega ^1_S$. We then define $\mathcal F_S$ by taking $T_{{\mathcal {F}}_S}$ to be the annihilator of $N_S$. Since $T_{{\mathcal {F}}}$ is locally free in a neighbourhood of the general point of *D* it follows that the rank of $T_{{\mathcal {F}}_S}$ is equal to the rank of $T_{{\mathcal {F}}}$.

The remaining claims then follow as in the proof of Proposition-Definition 3.7. We briefly sketch this now. Let $m>0$ be such that $m(K_{{\mathcal {F}}}+\Delta )$ is Cartier and set $L {:=} {\mathcal {O}}_X(m(K_{\mathcal F}+\Delta ))$. Since *D* is ${{\mathcal {F}}}$-invariant, the restriction of the morphism $\phi :(\Omega ^r_X)^{\otimes m} \rightarrow L$ to *D* factors through $\psi :(\Omega ^r_D)^{\otimes m} \rightarrow L\vert _D$. This lifts to a morphism ${\tilde{\psi }} :(\Omega ^r_S)^{\otimes m} \rightarrow n^*L$ by Proposition 3.1 (we remark that constructing this lift is local about any point of $x \in D$, and so by considering $\psi$ as a multi-derivation on ${\mathcal {O}}_{D, x}$, we see that the proof of Proposition 3.1 works equally well here).

We conclude by observing that around a general point of *S*, ${\tilde{\psi }}$ is the *m*-th tensor power of the Pfaff field $\Omega ^r_S \rightarrow {\mathcal {O}}_S(K_{{\mathcal {F}}_S})$ and so we deduce that there exists an effective divisor *B* such that $L|_S \cong {\mathcal {O}}_S(mK_{{\mathcal {F}}_S}+B)$. We conclude by taking $\textrm{Diff}_S({{\mathcal {F}}},\Delta ) {:=} \frac{1}{m}B$. $\square$

#### Remark 3.13

Set up as in Definition-Proposition 3.12. If $r=1$ then (3) is automatically satisfied since, by assumption, $K_{{{\mathcal {F}}}}$ is Cartier at the generic point of *D*.

Note moreover that, under the set-up of Proposition-Definition 3.7, if *D* is an ${{\mathcal {F}}}$-invariant integral subscheme of codimension one, then the construction of $(\mathcal F_S, \textrm{Diff}_S({{\mathcal {F}}},\Delta ))$ in Proposition-Definition 3.7 coincides with its construction in Proposition-Definition 3.12.

### Calculation of the different in some special cases

The following proposition computes the different in some special cases:

#### Proposition 3.14

Let *X* be a normal scheme, let $\mathcal F$ be a foliation of rank *r* on *X*, let $D \subset X$ be a reduced subscheme of codimension one such that either every component of *D* is ${{\mathcal {F}}}$-invariant or every component of *D* is not $\mathcal {F}$-invariant. Suppose that $K_{{\mathcal {F}}}$ and $\epsilon (D)D$ are ${\mathbb {Q}}$-Cartier. Let $n:S \rightarrow D$ be the normalisation, let ${{\mathcal {F}}}_S$ be the restricted foliation on *S* and let *P* be a prime divisor on *S*.

Then the following hold:

1. If *n*(*P*) is contained in ${{\,\textrm{Sing}\,}}\, {{\mathcal {F}}}$ then $m_P\textrm{Diff}_S({{\mathcal {F}}}) >0$.
2. Suppose that *D* is Cartier, $\epsilon (D) = 1$ and that $T_{{\mathcal {F}}}$ is locally free and generated by $\partial _1, \dots , \partial _r$. If *f* is a local parameter for *D* and $(\partial _1(f), \dots , \partial _r(f)) \subset I_{n(P)}$ then $m_P\textrm{Diff}_S({{\mathcal {F}}})>0$. Moreover, $m_P\textrm{Diff}_S({{\mathcal {F}}})$ is an integer. Conversely, suppose that *X* and *D* are smooth at the generic point of *n*(*P*), and that $m_P\textrm{Diff}_S({{\mathcal {F}}})>0$ then $(\partial _1(f), \dots , \partial _r(f)) \subset I_{n(P)}$.
3. Suppose that $\epsilon (D) = 0$ and that *n*(*P*) is not contained in ${{\,\textrm{Sing}\,}}\, {{\mathcal {F}}}$. Then $m_P\textrm{Diff}_S({{\mathcal {F}}}) = \frac{m-1}{m}\epsilon ({\mathcal {F}}_S, P)$ where *m* is a positive integer that divides the Cartier index of $K_{{\mathcal {F}}}$ at the generic point of *n*(*P*). If the index one cover associated to $K_{{\mathcal {F}}}$ is smooth in a neighbourhood of the preimage of the generic point of *n*(*P*), then *m* equals the Cartier index of $K_{{\mathcal {F}}}$ at the generic point of *n*(*P*).
4. Suppose that $\epsilon (D) = 1$ and that *X* has quotient singularities in a neighbourhood of the generic point of *n*(*P*). Then $m_P\textrm{Diff}_S({{\mathcal {F}}})$ is of the form $\frac{a+\epsilon ({\mathcal {F}}_S, P)(m-1)}{m}$ where $a \ge 0$ is an integer and where *m* divides the Cartier index of $K_{{\mathcal {F}}}$ at the generic point of *n*(*P*).

#### Proof

All these assertions are local about the generic point of *n*(*P*), so at any point we may freely replace *X* by a neighbourhood of the generic point of *n*(*P*).

Proof of item (1). Let us first suppose that $K_{{\mathcal {F}}}$ and $\epsilon (D)D$ are Cartier in a neighbourhood of *P*. By assumption, the morphism $\phi :\Omega ^r_X \rightarrow \mathcal O_X(K_{{\mathcal {F}}})$ takes values in $I{\mathcal {O}}_X(K_{{\mathcal {F}}})$ where *I* is an ideal sheaf, whose co-support contains *n*(*P*).

If $\epsilon (D) = 0$ then by Lemma 3.4 the lift of the restriction of $\phi$ to *D* factors through

$$\begin{aligned} \Omega ^r_S \rightarrow {\mathcal {O}}_S(n^*K_{{\mathcal {F}}} - P). \end{aligned}$$

If $\epsilon (D) = 1$ then, as in the proof of Lemma 3.5, we may define $\psi ':\Omega ^{r-1}_X \rightarrow {\mathcal {O}}_X(K_{{\mathcal {F}}}+D)$ by $\psi '{:=} \frac{\phi (df \wedge \cdot )}{f}$ for some choice *f* of a local parameter for *D*. Then $\psi '$ still factors through $I\mathcal O_X(K_{{\mathcal {F}}}+D)$ and so by Lemma 3.4 the lift of the restriction to *D* factors through

$$\begin{aligned} \Omega ^{r-1}_S \rightarrow {\mathcal {O}}_S(n^*(K_{{\mathcal {F}}}+D)- P). \end{aligned}$$

In both cases, we have that $m_P\textrm{Diff}_S({{\mathcal {F}}})\ge 1$, as required.

We now consider the general case. Let $q:V\rightarrow U$ be a quasi-étale cyclic cover, where *U* is a neighbourhood of the generic point of *P* in *X*, and such that $q^*K_{{{\mathcal {F}}}}$ and $q^*(\epsilon (D)D)$ are Cartier divisors. Let ${{\mathcal {F}}}'=q^{-1}\mathcal {F}$. Reference [18, Corollary 5.14] guarantees that *P* is contained in ${{\,\textrm{Sing}\,}}\, {{\mathcal {F}}}$ if and only $P'{:=} q^{-1}(P)$ is contained in ${{\,\textrm{Sing}\,}}\, {\mathcal {F}}'$. Let $S'$ denote the normalisation of $q^{-1}(D)$ and let ${{\mathcal {F}}}_{S'}$ and $\textrm{Diff}_{S'}({{\mathcal {F}}}')$ be respectively the restricted foliation and the different associated to ${\mathcal {F}}'$ on $S'$. We have a finite morphism $p:S' \rightarrow S$ and we have that

$$\begin{aligned} K_{{\mathcal {F}}_{S'}}+\textrm{Diff}_{S'}({{\mathcal {F}}}') = p^*(K_{\mathcal F_S}+\textrm{Diff}_S({{\mathcal {F}}})). \end{aligned}$$

Let *e* be the ramification index of *p* along $P'$. By applying [18, Lemma 3.4] (see also [10, Proposition 2.2]) we get an equality

1 $$\begin{aligned} m_{P'}\textrm{Diff}_{S'}({{\mathcal {F}}}') = em_P\textrm{Diff}(\mathcal {F}) - \epsilon ({{\mathcal {F}}}_S,P)(e-1). \end{aligned}$$

Since $m_{P'}\textrm{Diff}_{S'}({{\mathcal {F}}}')\ge 1$ this implies that $m_P\textrm{Diff}_S({{\mathcal {F}}}) >0$.

Proof of item (2). In this case, the Pfaff field $\phi :\Omega ^r_X \rightarrow {\mathcal {O}}_X(K_{{{\mathcal {F}}}})$ associated to ${\mathcal {F}}$ is given explicitly by $\omega \mapsto \omega (\partial _1\wedge \dots \wedge \partial _r)$ where $\omega$ is a local section of $\Omega ^r_X$. It follows that if $\eta$ is any local section of $\Omega ^{r-1}_X$ then

$$\begin{aligned} \phi (df \wedge \eta ) = \sum _{i=1}^r (-1)^i \partial _i(f) \eta (\partial _1 \wedge \dots \wedge \hat{\partial _i} \wedge \dots \wedge \partial _r). \end{aligned}$$

So if $(\partial _1(f), \dots , \partial _r(f)) \subset I_{n(P)}$, then Lemma 3.4 implies that the restricted Pfaff field $\Omega ^{r-1}_S \rightarrow n^*({\mathcal {O}}_X(K_{\mathcal F}+D))$ vanishes along *P*. Since $K_{{{\mathcal {F}}}}$ is Cartier, it follows that $m_P\textrm{Diff}_S({{\mathcal {F}}})$ is an integer.

Conversely, suppose that *D* and *X* are smooth at the generic point of *P* and there exists an *i* such that $\partial _i(f) \notin I_P$. Up to relabelling we may take $i = 1$ and after localising about the generic point of *P* we may assume $\partial _1(f)$ is a unit. For $j\ge 2$, we define

$$\begin{aligned} \partial _j' {:=} \partial _j - \frac{\partial _j(f)}{\partial _1(f)}\partial _1. \end{aligned}$$

We have that $\partial _1, \partial _2', \dots , \partial _r'$ generate $T_{{\mathcal {F}}}$ in a neighbourhood of the generic point of *P* and so, up to replacing $\partial _j$ by $\partial '_j$, we may freely assume that $\partial _j(f) = 0$ for $j \ge 2$. A direct calculation as above shows that the restricted Pfaff field $\Omega ^{r-1}_D \rightarrow {\mathcal {O}}_D(K_{{\mathcal {F}}}+D)$ does not vanish along *P*, as required.

Proof of item (3). Suppose for the moment that $K_{{\mathcal {F}}}$ is Cartier. In this case, the natural map $\Omega ^r_D \rightarrow {\mathcal {O}}_D(K_{{\mathcal {F}}})$ vanishes along *P* if and only if $\Omega ^r_X \rightarrow {\mathcal {O}}_X(K_{{\mathcal {F}}})$ vanishes along *P*, and so $m_P\textrm{Diff}_S({{\mathcal {F}}}) = 0$ as required.

We now handle the case where $K_{{\mathcal {F}}}$ is $\mathbb {Q}$-Cartier. After possibly shrinking *X*, we consider the index one cover $q:X' \rightarrow X$ associated to $K_{{\mathcal {F}}}$ and let $S'$ be the normalisation of $q^{-1}(D)$. Note that the ramification index of $S' \rightarrow S$ along $P' := q^{-1}(P)$ divides the Cartier index of $K_{{\mathcal {F}}}$ and that if ${{\mathcal {F}}}':= q^{-1}{{\mathcal {F}}}$ then [18, Corollary 5.14] implies that $P'$ is not contained in ${{\,\textrm{Sing}\,}}\, {{\mathcal {F}}}'$. We may then argue as in the proof of Item (1) and use Eq. (1) to conclude.

To check the last claim, it suffices to consider the case where $\epsilon ({\mathcal {F}}_S, P) = 1$. After possibly replacing *X* by a neighbourhood of the generic point of *P*, we may assume that the index one cover $q:X' \rightarrow X$ associated to $K_{\mathcal {F}}$ is such that $X'$ is smooth and it is totally ramified along *P*. In particular, we may assume that $D':= q^{-1}(D)$ is connected. Let *m* be the Cartier index of $K_{{\mathcal {F}}}$ and let ${{\mathcal {F}}}':= q^{-1}{{\mathcal {F}}}$. Since $T_{{\mathcal {F}}'}$ is reflexive, by [22, Corollary 1.4] it is locally free away from a subset of codimension at least three in *X*. Thus, we may assume that $T_{{\mathcal {F}}'}$ is locally free. Let $P':= q^{-1}(P)$.

We claim that $D'$ is normal and irreducible. Assuming the claim we see that the ramification index of the induced morphism $D' \rightarrow D$ along $P'$ is *m*, and we may conclude by Eq. (1).

To prove the claim, first recall that $D'$ is connected. Suppose that $D'$ is not smooth in a neighbourhood of $P'$. By [33, Theorem 5] for any local generator $\partial \in T_{{\mathcal {F}}'}$ we have that $\partial (I_{{{\,\textrm{Sing}\,}}{D'}}) \subset I_{{{\,\textrm{Sing}\,}}{D'}}$. Thus if we denote by ${\mathcal {F}}_{S'}^\circ$ the integrable distribution induced on $S'$, where $n':S'\rightarrow D'$ is the normalisation and whose existence is guaranteed by Lemma 3.5, then for any local generator $\partial \in T_{{\mathcal {F}}_{S'}^\circ }$ we have $\partial (I_{P'}) \subset I_{P'}$. Since we assumed that *P* is not ${\mathcal {F}}_S$-invariant, it follows that $P'$ is not ${\mathcal {F}}_{S'}$-invariant and so there is a local generator $\delta \in T_{{\mathcal {F}}_{S'}}$ such that in a neighbourhood of the generic point of $P'$, $\delta (I_{P'})$ is not contained in $I_{P'}$. On the other hand, $P'$ is ${\mathcal F_{S'}^\circ }$-invariant and so it follows that in a neighbourhood of the generic point of $P'$ there is some local section $f \in I_{P'}$ such that $f\delta$ is a local generator of $T_{\mathcal F_{S'}^\circ }$. Thus, some local generator of $T_{\mathcal F_{S'}^\circ }$ must vanish along $P'$, which in turn implies that some local generator of $T_{{\mathcal {F}}'}$ must vanish along $n'(P')$ and, in particular, $n'(P')$ is contained in ${{\,\textrm{Sing}\,}}\, {{\mathcal {F}}}'$. [18, Corollary 5.14] implies that *n*(*P*) is contained in ${{\,\textrm{Sing}\,}}\, {{\mathcal {F}}}$, contrary to our hypothesis.

Proof of item (4). As in the proof of item (1) using Eq. (1) we may freely replace *X* by a quasi-étale cover and so may assume that *X* is smooth. By [22, Corollary 1.4] we may assume that $T_{{\mathcal {F}}}$ is locally free. We may then conclude by Item (2). $\square$

### Adjunction of singularities

#### Lemma 3.15

Let *X* be a smooth variety, let ${\mathcal {F}}$ be a foliation of rank *r* on *X* and let *D* be a smooth divisor on *X* such that *D* is not ${\mathcal {F}}$-invariant. Let $Z \subset D$ be a codimension one subvariety in *D*.

Then there exists a log resolution $\mu :X' \rightarrow X$ of (*X*, *D*) such that if we write $(K_{{\mathcal {F}}'}+D')\vert _{D'} = K_{{\mathcal {F}}_{D'}}+\textrm{Diff}_{D'}({{\mathcal {F}}})$ where $D' = \mu _*^{-1}D$, ${\mathcal {F}}' = \mu ^{-1}{\mathcal {F}}$ and $\mathcal F_{D'}$ is the restricted foliation on $D'$, then $Z'$ is not contained in the support of $\textrm{Diff}_{D'}({{\mathcal {F}}}')$, where $Z'\subset D'$ is the strict transform of *Z* through the induced morphism $D'\rightarrow D$. In particular, ${{\mathcal {F}}}'$ is smooth in a neighbourhood of the generic point of $Z'$.

#### Proof

We may freely shrink about the generic point of *Z*, and so we may assume that $T_{{\mathcal {F}}}$ is locally free [22, Corollary 1.4], and generated by $\partial _1, \dots , \partial _r$.

We will construct a model $\mu :X' \rightarrow X$ such that, using the same notation as in the statement of the Lemma, there is a vector field $\partial \in T_{{\mathcal {F}}'}$ such that $\partial (I_{D'})$ is not contained in $I_{Z'}$. By (1) and (2) of Proposition 3.14 this will be our desired model.

Up to relabelling we may assume that $\partial _1 \in T_{{\mathcal {F}}}$ does not leave *D* invariant.

Shrinking to a neighbourhood of the generic point of *Z* we may find coordinates $(x, y, z_1, \dots , z_q) = (x, y, {\underline{z}})$ where $\dim X = q+2$ such that $D = \{x = 0\}, Z = \{x = y = 0\}$ and

$$\begin{aligned} \partial _1 = a(x, y, {\underline{z}})\partial _x+b(x, y, {\underline{z}})\partial _y+\sum _{j = 1}^q c_j(x, y, {\underline{z}})\partial _{z_j} \end{aligned}$$

where $a,b,c_1,\dots ,c_q\in {\mathcal {O}}_{X, Z}$. If *a* is a unit in ${\mathcal {O}}_{X, Z}$ then we may take $X' = X$ and we are done, so we may assume that $a \in (x, y)$. Since *D* is not $\partial _1$-invariant we also see that $a \notin (x)$ and so (up to multiplying by a unit in ${\mathcal {O}}_{X, Z}$) we may write $a = y^k+x\alpha (x, y, {\underline{z}})$ where $k\ge 0$ and for some function $\alpha \in {\mathcal {O}}_{X, Z}$. If $k = 0$, then *a* is a unit and we are done. Thus, we assume that $k \ge 1$. Note that *k* is the order of tangency between $\partial _1$ and *D* along *Z*. We will show that after a finite sequence of blow ups we can reduce the order of tangency between the strict transform of *D* and the pullback of the foliation ${\mathcal {F}}_1$ defined by $\partial _1$. Assuming this can be done, then we may conclude by arguing by induction on *k*.

Consider the local chart of the blow up $b_1:X_1 \rightarrow X$ in *Z* given by $x = x_1y_1, y = y_1, z_i = z_i$. In this chart, the strict transform of *D* is given by $\{x_1 = 0\}$ and the transform $Z_1$ of *Z* (considered as subvariety of *D*) is given by $\{x_1 = y_1 = 0\}$. The pullback of $\partial _1$ as a meromorphic vector field is

$$\begin{aligned} \left[ (y_1^{k-1}+x_1\alpha (x_1y_1, y_1, {\underline{z}})-\frac{x_1}{y_1}b(x_1y_1, y_1, {\underline{z}})\right] \partial _{x_1}+ b(x_1y_1, y_1, {\underline{z}})\partial _{y_1}+ \dots . \end{aligned}$$

If the pullback of $\partial _1$ is in fact holomorphic, then in a neighbourhood of the transform $Z_1$ of *Z*, $b_1^{-1}{\mathcal {F}}$ is defined by a vector field of the form $(y_1^j+x_1\alpha _1)\partial _{x_1} +\dots$ where $j <k$ and for some function $\alpha _1\in {\mathcal {O}}_{X_1, Z_1}$. Otherwise, $b_1^{-1}{\mathcal {F}}$ is defined by a vector field of the form

$$\begin{aligned} \delta = (y_1^k+x_1\alpha _1)\partial _{x_1}+y_1\beta _1\partial _{y_1}+\dots \end{aligned}$$

for some functions $\alpha _1,\beta _1\in {\mathcal {O}}_{X_1, Z_1}$. It is easy to check that if $b_2:X_2 \rightarrow X_1$ is the blow up in $Z_1$, then the pullback of $\delta$ is holomorphic. In particular, if we take the following coordinates on the blow up $x_1 = x_2y_2, y_1 = y_2, z_i = z_i$, then in a neighbourhood of $Z_2$, the transform of $Z_1$, $b_2^{-1}b_1^{-1}{\mathcal {F}}_1$ is defined by a vector field of the form $(y_2^j+x_2\alpha _2)\partial _{x_2}+\dots$ where $j<k$ and for some function $\alpha _2\in {\mathcal {O}}_{X_2, Z_2}$ Thus, after at most two blow ups centred over *Z* we see that we can reduce the order of tangency between the strict transform of *D* and the pullback of ${\mathcal {F}}_1$, as required. $\square$

#### Theorem 3.16

Let *X* be a normal variety, let ${\mathcal {F}}$ be a foliation of rank *r* on *X* and let *D* be a prime divisor which is not ${\mathcal {F}}$-invariant. Let $0 \le \Delta = D+\Delta '$ be a ${\mathbb {Q}}$-divisor on *X* such that *D* is not contained in the support of $\Delta '$. Let $Z\subset X$ be a subvariety such that $Z\cap D$ is of codimension at least two in *D* and such that $K_{{{\mathcal {F}}}}$ and *D* are ${\mathbb {Q}}$-Cartier on $X{\setminus } Z$. Suppose that $({\mathcal {F}}, \Delta )$ is canonical (resp. log canonical, resp. terminal, resp. log terminal). Let $S \rightarrow D$ be the normalisation and let $\Delta _S := \textrm{Diff}_S({{\mathcal {F}}},\Delta ')$.

Then $({\mathcal {F}}_S, \Delta _S)$ is canonical (resp. log canonical, resp. terminal, resp. log terminal).

#### Proof

Pick any divisor $E_S$ on some birational model $S' \rightarrow S$. Let $\mu :Y \rightarrow X$ be any log resolution of $(X, \Delta )$ (we emphasise that this not necessarily a log resolution of ${\mathcal {F}}$) which extracts a divisor *E* such that $E \cap S_Y = E_S$ where $S_Y := \mu _*^{-1}D$. Let ${\mathcal {F}}_Y := \mu ^{-1}{\mathcal {F}}$ and $\Delta _Y := \mu _*^{-1}\Delta$.

By Lemma 3.15, perhaps replacing *Y* by a higher model we may assume that if we write $(K_{\mathcal F_Y}+S_Y)\vert _{S_Y} \sim K_{{\mathcal {F}}_{S_Y}}+\Theta _{S_Y}$ then $\Theta _{S_Y}$ does not contain $E_S$ in its support.

By assumption $a(E, {\mathcal {F}}, \Delta ) \ge 0$ (resp. $\ge -\epsilon ({\mathcal {F}}_Y, E)$, etc.) and so we see that $a(E_S, {\mathcal {F}}_S, \Delta _S) \ge 0$ (resp. $\ge -\epsilon ({\mathcal {F}}_Y, E)$, etc.). To conclude it suffices to show that if $a(E, \mathcal F, \Delta ) < 0$ then $\epsilon ({\mathcal {F}}_{S_Y}, E_S) = 1$. Suppose for sake of contradiction that $\epsilon ({\mathcal {F}}_{S_Y},E_S) = 0$. By Lemma 3.15 we see that $E_S \subset Y$ is not contained in ${{\,\textrm{Sing}\,}}\, {{\mathcal {F}}_Y}$ and so shrinking about a general point of $E_S$ we may assume that ${\mathcal {F}}_Y$ is smooth, and that $\Delta _Y = S_Y$. If $a(E, {\mathcal {F}}, \Delta )<0$ then a direct calculation shows that the blow up of *Y* along $E_S$ extracts an invariant divisor *F* such that

$$\begin{aligned} a(F,{\mathcal {F}},\Delta )=a(F,{\mathcal {F}}_Y, S_Y-a(E, {\mathcal {F}}, \Delta )E)<0, \end{aligned}$$

contradicting the hypothesis that $({\mathcal {F}}, \Delta )$ is log canonical. Thus, our claim follows. $\square$

#### Corollary 3.17

Let *X* be a potentially klt variety, let ${\mathcal {F}}$ be a foliation of rank *r* on *X* and let *D* be a divisor which is not ${\mathcal {F}}$-invariant. Let $0 \le \Delta = D+\Delta '$ be a ${\mathbb {Q}}$-divisor on *X* such that *D* is not contained in the support of $\Delta '$. Suppose that *D*, $K_{{\mathcal {F}}}+\Delta$ and $K_X+\Delta$ are ${\mathbb {Q}}$-Cartier. Let $n:S \rightarrow D$ be the normalisation and let $\textrm{Diff}_S(X, \Delta ')$ be the classical different associated to $(X, \Delta )$ on *S*.

Then we have an inequality of differents

$$\begin{aligned} \textrm{Diff}_S({\mathcal {F}}, \Delta ')_{\mathrm{n-inv}} \ge \textrm{Diff}_S(X, \Delta ')_{\mathrm{n-inv}}. \end{aligned}$$

#### Proof

To prove the claim it suffices to work in the neighbourhood of the generic point of a divisor $P \subset S$, which is not $\mathcal F_S$-invariant. Since *X* is potentially klt, it has quotient singularities in a neighbourhood of the generic point of *P*. Arguing as in the proof of item (1) of Proposition 3.14 we may freely replace *X* by a finite cover, and so we may assume that *X* is smooth.

Suppose that $\pi :{\overline{X}} \rightarrow X$ is a log resolution of $(X, D+\textrm{Supp}\, \Delta )$. We may write

$$\begin{aligned} K_{\overline{{\mathcal {F}}}}+{\overline{\Delta }}+ {\overline{D}}+E = \pi ^*(K_{{\mathcal {F}}}+\Delta +D) \end{aligned}$$

and

$$\begin{aligned} K_{{\overline{X}}}+{\overline{\Delta }}+{\overline{D}}+E' = \pi ^*(K_{{\mathcal {F}}}+\Delta +D) \end{aligned}$$

where $E, E'$ are $\pi$-exceptional, $\overline{{\mathcal {F}}} := \pi ^{-1}{\mathcal {F}}$, ${\overline{D}}:= \pi _*^{-1}D$ and ${\overline{\Delta }} := \pi _*^{-1}\Delta$. By [34, Corollary 3.3] we have that $E'\le E$. Let *S* be the normalisation of *D* and let $\mu :{\overline{D}} \rightarrow S$ be the induced morphism.

By Remark 3.10 we have that $\textrm{Diff}_{\overline{D}}(\overline{{{\mathcal {F}}}},{\overline{\Delta }})\ge \overline{\Delta }|_{{{\overline{D}}}}$. Thus, if $\textrm{Diff}_{\overline{D}}({\overline{X}},{\overline{\Delta }})$ denotes the classical different on ${\overline{X}}$, then $\textrm{Diff}_{\overline{D}}({\overline{X}},{\overline{\Delta }})= {{\overline{\Delta }}}|_{{{\overline{D}}}}$ and by Remark 3.11 we have

$$\begin{aligned} \begin{aligned} \textrm{Diff}_S({{\mathcal {F}}},\Delta )&= \mu _*(\textrm{Diff}_{{{\overline{D}}}}(\overline{{{\mathcal {F}}}},{\overline{\Delta }})+E|_{{\overline{D}}}) \\&\ge \mu _*(\textrm{Diff}_{\overline{D}}({\overline{X}},{\overline{\Delta }})+E'|_{{{\overline{D}}}})=\textrm{Diff}_S(X,\Delta ). \end{aligned} \end{aligned}$$

Thus, our claim follows. $\square$

#### Lemma 3.18

Let *X* be a potentially klt variety, let ${\mathcal {F}}$ be an algebraically integrable foliation of rank *r* on *X* which is induced by a morphism $f:X \rightarrow Z$ and let *D* be a divisor which is not ${\mathcal {F}}$-invariant. Let $0 \le \Delta = D+\Delta '$ be a ${\mathbb {Q}}$-divisor on *X* such that *D* is not contained in the support of $\Delta '$. Suppose that *D*, $K_{{\mathcal {F}}}+\Delta$ and $K_X+\Delta$ are ${\mathbb {Q}}$-Cartier. Let $n:S \rightarrow D$ be the normalisation and let $\textrm{Diff}_S(X, \Delta ')$ be the classical different associated to $(X, \Delta )$ on *S*. Suppose that $({\mathcal {F}}, \Delta )$ is log canonical.

Then

$$\begin{aligned} \textrm{Diff}_S({\mathcal {F}}, \Delta ') = \textrm{Diff}_S(X, \Delta ')_{\mathrm{n-inv}}. \end{aligned}$$

#### Proof

By Corollary 3.17 we have an inequality

$$\begin{aligned} \textrm{Diff}_S({\mathcal {F}}, \Delta ') \ge \textrm{Diff}_S(X, \Delta ')_{\mathrm{n-inv}}. \end{aligned}$$

By Theorem 3.16 we have that $({\mathcal {F}}_S, \textrm{Diff}_S({\mathcal {F}}, \Delta '))$ is log canonical and by Lemma 2.5 it follows that $\textrm{Diff}_S({\mathcal {F}}, \Delta ')$ has no ${\mathcal {F}}_S$-invariant components. Thus, it suffices to show that if $P \subset S$ is a divisor which is not $\mathcal F_{S}$-invariant then the coefficient of *P* in each of the differents is the same.

Since ${\mathcal {F}}$ is induced by the morphism $f:X \rightarrow Z$, ${\mathcal {F}}_{S}$ is induced by the restricted morphism $(f\circ n):S \rightarrow Z$. Since *P* is not $\mathcal F_{S}$-invariant, *P* dominates *Z*. Let $\mu :X' \rightarrow X$ be any birational model and *E* be any $\mu$-exceptional divisor centred on *n*(*P*). Let us define

$$\begin{aligned} G := (K_{\mu ^{-1}\mathcal F}+\mu _*^{-1}\Delta -\mu ^*(K_{{\mathcal {F}}}+\Delta ) ) - (K_{X'}+\mu _*^{-1}\Delta -\mu ^*(K_X+\Delta )). \end{aligned}$$

Note that *G* is $\mu$-exceptional. In a neighbourhood of the generic fibre of $X' \rightarrow Z$ we have that $K_{\mu ^{-1}{\mathcal {F}}} \equiv K_{X'}$, and so *G* is $\mu$-numerically trivial in a neighbourhood of the generic fibre of $X' \rightarrow Z$. By the negativity lemma (cf. [25, Lemma 3.39]) in this neighbourhood $G = 0$. In particular, since *E* dominates *Z*, we have $a(E, {\mathcal {F}}, \Delta ) = a(E, X, \Delta )$. By Remark 3.11 we may then conclude that

$$\begin{aligned} m_P\textrm{Diff}_S({\mathcal {F}}, \Delta ') = m_P\textrm{Diff}_S(X, \Delta ') \end{aligned}$$

as required. $$\square $$

#### Corollary 3.19

Let *X* be a potentially klt variety, let ${\mathcal {F}}$ be a foliation on *X* and let ${\mathcal {H}} \subset {\mathcal {F}}$ be an algebraically integrable subfoliation which is induced by a morphism and let *D* be a divisor on *X* which is not ${\mathcal {H}}$-invariant. Let $0 \le \Delta = D+\Delta '$ be a ${\mathbb {Q}}$-divisor on *X* such that *D* is not contained in the support of $\Delta '$. Suppose that *D*, $K_{{\mathcal {F}}}+\Delta$ and $K_X+\Delta$ are ${\mathbb {Q}}$-Cartier and that $({\mathcal {H}}, \Delta )$ is log canonical. Let $n:S \rightarrow D$ be the normalisation.

Then

$$\begin{aligned} \textrm{Diff}_S({\mathcal {F}}, \Delta ') \ge \textrm{Diff}_S({\mathcal {H}}, \Delta '). \end{aligned}$$

#### Proof

We first remark that *D* is not ${\mathcal {F}}$-invariant and if $P\subset S$ is a prime divisor which is not ${{\mathcal {H}}}_S$-invariant, then it is also not ${{\mathcal {F}}}_S$-invariant. Thus, the claim is a direct consequence of Corollary 3.17 combined with Lemma 3.18. $\square$

### Additional remarks

#### Failure of adjunction on singularities for invariant divisors

Let ${{\mathcal {F}}}$ be a log canonical foliation on a smooth variety *X* and let *D* be a smooth ${{\mathcal {F}}}$-invariant divisor on *X*. Then in general $({\mathcal {F}}_D, \textrm{Diff}_D(\mathcal {F}))$ is not log canonical, as the following example shows:

##### Example 3.20

Let $X = {\mathbb {A}}^3$ with coordinates *x*, *y*, *z* and let $\mathcal F$ be the rank one foliation on *X* defined by the vector field

$$\begin{aligned} \partial := x^2\partial _x+y^2\partial _y+z\partial _z. \end{aligned}$$

By [30, Fact I.ii.4] ${\mathcal {F}}$ has log canonical singularities since its semi-simple part $z\partial _z$ is non-zero. Set $D = \{z = 0\}$. Then *D* is ${{\mathcal {F}}}$-invariant and $\mathcal F_D$ is generated by $x^2\partial _x+y^2\partial _y$ whose semi-simple part is zero and by [30, Fact I.ii.4] again, it is not log canonical.

#### Failure of Bertini’s theorem

Let *X* be a smooth variety and let ${\mathcal {F}}$ be a foliation with canonical singularities. Let *A* be an ample Cartier divisor. In general it is not the case that we may find $0 \le D \sim _{\mathbb {Q}} A$ such that $({\mathcal {F}}, D)$ is log canonical, as the following example shows (see also [1, pag. 12]). Let $\pi :X_0:={\mathbb {P}}^2\times {\mathbb {P}}^1\rightarrow {\mathbb {P}}^2$ be the projection, let $\ell \subset X_0$ be a smooth curve such that $\pi (\ell )$ is a line and let *X* be the blow up of $X_0$ along $\ell$. Then the foliation ${{\mathcal {F}}}$ induced by the morphism $X\rightarrow {\mathbb {P}}^2$ admits canonical singularities, but $({{\mathcal {F}}},D)$ is not log canonical for any ample ${\mathbb {Q}}$-divisor $D\ge 0$.

Moreover, the same example shows that it is in general not possible to choose *D* to be reduced and irreducible and so that $\textrm{Diff}_S({{\mathcal {F}}}) = 0$, where $S\rightarrow D$ is the normalisation.

#### Other definitions of foliation

Occasionally in the literature a foliation is defined to be a quotient of the cotangent sheaf. From the perspective of defining an adjunction formula (especially on singular varieties) our definition seems to be more appropriate.

The $S_2$ condition likewise seems to be important. On non-normal varieties there exist quotients of the cotangent sheaf which do not seem to correspond to reasonable foliations. Consider the following example. Let $X:= \{x = y = 0\} \cup \{z = w = 0\} \subset {\mathbb {A}}^4$, let $\omega$ be the restriction to *X* of the 1-form $dz+xdy+ydx$ and consider the quotient $\Omega ^1_X \rightarrow \Omega ^1_X/(\omega )$. There is no way to lift this quotient to a rank one foliation on a neighbourhood of (0, 0, 0, 0), however, for a foliation one would expect that this should always be possible.

## Cone theorem for rank one foliated pairs

Throughout this section we assume that all our varieties are defined over ${\mathbb {C}}$.

The cone theorem for rank one foliations was initially considered in [7], and a refined version has appeared in [28]. The goal of this section is to present a more general version which is more suitable to run the MMP.

In order to study curves which are contained in the singular locus of a foliation we first need some preliminary results. Indeed, the definition of the singular locus of a rank one foliation presented in [28] is slightly different than the one given above. We recall McQuillan’s definition now.

Let *X* be a normal variety, let ${\mathcal {F}}$ be a rank one foliation on *X* such that $K_{{{\mathcal {F}}}}$ is ${\mathbb {Q}}$-Cartier. Let $x \in X$ be a point and let *U* be an open neighbourhood of *x*. Up to replacing *U* by a smaller neighbourhood we may find an index one cover $\sigma :U' \rightarrow U$ associated to $K_{{\mathcal {F}}}$ and such that $\sigma ^{-1}{\mathcal {F}}$ is generated by a vector field $\partial$.

We say that ${\mathcal {F}}$ is **singular in the sense of McQuillan** at $x \in X$ provided there exists an embedding $U' \rightarrow M$ where *M* is a smooth variety and a lift ${\tilde{\partial }}$ of $\partial$ to a vector field on *M* such that ${\tilde{\partial }}$ vanishes at $\sigma ^{-1}(x)$. We denote by ${{\,\textrm{Sing}\,}}^+{{\mathcal {F}}}$ the locus of points $x \in X$ where ${\mathcal {F}}$ is singular in the sense of McQuillan. Note that ${{\,\textrm{Sing}\,}}^+{{\mathcal {F}}}$ does not depend on the choice of $U'$ and it is a closed subset of *X*.

On a smooth variety it is easy to see that ${{\,\textrm{Sing}\,}}^+{{\mathcal {F}}} = {{\,\textrm{Sing}\,}}{{\mathcal {F}}}$, but in general it is unclear if these two notions of singularity agree. We do, however, have the following inclusion of singular loci:

### Lemma 4.1

Let *X* be a normal variety, let ${\mathcal {F}}$ be a rank one foliation on *X* such that $K_{{{\mathcal {F}}}}$ is ${\mathbb {Q}}$-Cartier.

Then $${{\,\textrm{Sing}\,}}{\mathcal {F}} \subset {{\,\textrm{Sing}\,}}^+{{\mathcal {F}}}$$.

### Proof

By [18, Corollary 5.14] we may freely replace *X* by an index one cover of $K_{{\mathcal {F}}}$ and so we may assume that $K_{{\mathcal {F}}}$ is Cartier, in which case the Lemma is easy. $\square$

### Lemma 4.2

Let *X* be a normal variety, let ${\mathcal {F}}$ be a rank one foliation on *X* such that $K_{{{\mathcal {F}}}}$ is ${\mathbb {Q}}$-Cartier. Let $V \subset X$ be an irreducible subvariety such that *V* is not contained in ${{\,\textrm{Sing}\,}}^+\, {\mathcal {F}}$. Assume that there exists an index one cover $\sigma :X' \rightarrow X$ associated to $K_{{\mathcal {F}}}$ and let $\mathcal {F}':= \sigma ^{-1}{{\mathcal {F}}}$.

Then *V* is ${\mathcal {F}}$-invariant if and only if $V':= \sigma ^{-1}(V)$ is ${\mathcal {F}}'$-invariant. Moreover, if *V* is invariant and if we denote by ${\mathcal {F}}_V$ (resp. $\mathcal F'_{V'}$) the restricted foliation on *V* (resp. $V'$), then $(\sigma |_{V'})^{-1} {\mathcal {F}}_V = {\mathcal {F}}'_{V'}$.

### Proof

We first remark that if $\sigma$ is étale in a neighbourhood of the generic point of *V*, then both points of the lemma are clear.

Suppose that *V* is ${\mathcal {F}}$-invariant. In this case, by definition it follows that $K_{{\mathcal {F}}}$ is Cartier in a neighbourhood of the generic point of *V* and, therefore, $\sigma$ is étale in a neighbourhood of the generic point of *V*, in which case we may conclude.

Suppose that $\sigma ^{-1}(V)$ is ${\mathcal {F}}'$-invariant. Let $x \in \sigma ^{-1}(V)$ be a general point. By [7, Lemma I.2.1] there exists an analytic neighbourhood *U* of *x* and a holomorphic submersion $p:U \rightarrow Z$ such that $T_{\mathcal F'}\vert _U = T_{U/Z}$. After possibly shrinking *U*, we may assume that $\sigma$ is a Galois cover with Galois cover $G=\mathbb Z/m{\mathbb {Z}}$. Since the fibre of *p* are invariant with respect to *G*, it follows that the action of *G* descends to *Z*.

After possibly shrinking *U* further, we may write $U \cong \mathbb D \times Z$ where ${\mathbb {D}}$ is the unit disc and *p* is given by projection onto the second coordinate. Moreover, we may assume that *G* acts on ${\mathbb {D}}$ so that the diagonal action of *G* on ${\mathbb {D}} \times Z$ coincides with the action on *U*. In particular, if *t* is a coordinate on ${\mathbb {D}}$, then $T_{\mathcal F'}$ is defined by $\partial _t$ and the Galois group acts on *t* by $t \mapsto \xi ^{-1} t$ where $\xi$ is a primitive *m*-th root of unity. It follows that the ramification locus of $\sigma$ is of the form $\{t = f_1 = \dots = f_r = 0\}$, for some functions $f_1,\dots ,f_r\in {\mathcal {O}}_X$.

Since ${{\mathcal {F}}}'$ is defined by $\partial _{t}$, any $\mathcal F'$-invariant variety is locally of the form $p^{-1}(W)$ where $W \subset Z$ is a subvariety. Thus, no invariant subvariety is contained in the ramification locus of $\sigma$, and so $\sigma$ is étale at the generic point of *V* and we may conclude. $\square$

### Lemma 4.3

Let *X* be a normal variety, let ${\mathcal {F}}$ be a rank one foliation on *X* such that $K_{{{\mathcal {F}}}}$ is ${\mathbb {Q}}$-Cartier. Let $Z \subset X$ be a subvariety which is not ${{\mathcal {F}}}$-invariant and is not contained in ${{\,\textrm{Sing}\,}}^+\, {\mathcal {F}}$.

Then ${{\mathcal {F}}}$ is terminal at the generic point of *Z*.

### Proof

The problem is local about a general point $z \in Z$, so we are free to shrink about a general point of *Z*. By [30, Corollary III.i.5] and Lemma 4.2, we may therefore replace *X* by the index one cover associated to $K_{{\mathcal {F}}}$, and so we may assume that $K_{{\mathcal {F}}}$ is Cartier. The result then follows from [30, Fact III.i.1]. $\square$

### Lemma 4.4

Let $p:X\rightarrow Y$ be a smooth morphism between normal analytic varieties and let ${{\mathcal {F}}}$ be the foliation on *X* induced by *p*. Let $\Delta \ge 0$ be a ${\mathbb {Q}}$-Cartier ${\mathbb {Q}}$-divisor on *X*. Let $0\in Y$ and let $D_0:= p^{-1}(0)$.

Then $({{\mathcal {F}}},\Delta )$ is log canonical in a neighbourhood of $D_0$ if and only if $D_0$ is not contained in the support of $\Delta$ and $(D_0,\Delta _0)$ is log canonical, where $\Delta _0 := \Delta |_{D_0}$.

### Proof

Note that if $D_0$ is contained in the support of $\Delta$, $\dim Y \ge 2$ and $q:{{\overline{X}}}\rightarrow X$ is the blow up of *X* along $D_0$ with exceptional divisor *E* then *E* is $q^{-1}\mathcal {F}$-invariant and $a(E,{{\mathcal {F}}},\Delta )<0=\epsilon (E)$. Thus, $(\mathcal {F},\Delta )$ is not log canonical around $D_0$. If $\dim Y = 1$, and $D_0$ is contained in the support of $\Delta$ then $({\mathcal {F}}, \Delta )$ is not log canonical by Lemma 2.5. Therefore we may assume that $D_0$ is not contained in the support of $\Delta$.

Let $\beta :Y'\rightarrow Y$ be a resolution of *Y* and let $X' := X\times _{Y} Y'$. Let $\alpha :X'\rightarrow X$ and $p':X'\rightarrow Y$ be the induced morphisms. Then $p'$ is a smooth morphism and if ${{\mathcal {F}}}'$ is the foliation induced by $p'$, it follows that ${{\mathcal {F}}}'=\alpha ^{-1}{{\mathcal {F}}}$. Let $\Delta ' := \alpha _*^{-1}\Delta$. Then $K_{{{\mathcal {F}}}'}+\Delta '=\alpha ^*(K_{\mathcal {F}}+\Delta )$.

For any $y\in \beta ^{-1}(0)$, let $D_y := p'^{-1}(y)$ be the corresponding fibre. Note that $D_y$ is not contained in the support of $\Delta '$ and if $\Delta _y := \Delta '|_{D_y}$ then $(D_y,\Delta _y)\cong (D_0,\Delta _0)$.

Pick $y\in \beta ^{-1}(0)$ and let $\Sigma$ be a reduced divisor on $Y'$ such that $(Y',\Sigma )$ is log smooth and *y* is a zero-dimensional stratum of $\Sigma$. We claim that $(\mathcal {F}',\Delta ')$ is log canonical in a neighbourhood of $D_y$ if and only if $(X',\Delta '+p'^*\Sigma )$ is. Indeed, if $\gamma :X''\rightarrow X'$ is a birational morphism then, as in the proof of [1, Lemma 3.1], we have that

$$\begin{aligned} K_{X''}+\gamma _*^{-1}(p'^*\Sigma )=\gamma ^*(K_{X'}+p'^*\Sigma )+F \end{aligned}$$

and

$$\begin{aligned} K_{\gamma ^{-1}{\mathcal {F}}'} = \gamma ^*K_{{{\mathcal {F}}}'}+G \end{aligned}$$

where *F*, *G* are $\gamma$-exceptional divisors such that $G=F+\sum (1-\epsilon (E))E$, where the sum runs over all the $\gamma$-exceptional prime divisors. Thus, our claim follows.

By inductively applying inversion of adjunction (see [20, Theorem 1.1]), we have that $(X',\Delta '+p'^*\Sigma )$ is log canonical in a neighbourhood of $D_y$ if and only if $(D_y,\Delta _y)$ is log canonical. Thus, we have that $(\mathcal {F},\Delta )$ is log canonical (resp. log terminal) in a neighbourhood of $D_0$ if and only if $(D_0,\Delta _0)$ is log canonical (resp. log terminal).$\square$

### Lemma 4.5

Let *X* be a normal variety and let $\mathcal F$ be a rank one foliation on *X* such that $K_{{\mathcal {F}}}$ is ${\mathbb {Q}}$-Cartier. Let $Z \subset X$ be a subvariety which is not ${{\mathcal {F}}}$-invariant and it is not contained in ${{\,\textrm{Sing}\,}}^+\, \mathcal F$. Let *W* be a (possibly analytic) ${\mathcal {F}}$-invariant subvariety which contains *Z* with $\dim W = \dim Z+1$. Let $n:S \rightarrow W$ be the normalisation and let $\Delta \ge 0$ be a ${\mathbb {Q}}$-Cartier ${\mathbb {Q}}$-divisor on *X* which does not contain *W* in its support.

Then

1. there exists $\lambda >0$ such that $({\mathcal {F}}, \lambda \Delta )$ is terminal at the generic point of *Z*;
2. if $({{\mathcal {F}}},\Delta )$ is log canonical in a neighbourhood of the generic point of *Z* and ${\mathcal {F}}_S$ is the restricted foliation, then $({\mathcal {F}}_S, \textrm{Diff}_S(\mathcal {F},\Delta ))$ is log canonical in a neighbourhood of the generic point of $n^{-1}(Z)$.

### Proof

The problem is local about a general point $z \in Z$, so we are free to shrink about a general point of *Z*. By [30, Corollary III.i.5] and Lemma 4.2, we may therefore replace *X* by the index one cover associated to $K_{{\mathcal {F}}}$, and so we may assume that $K_{{\mathcal {F}}}$ is Cartier.

We may also assume that *Z* does not intersect ${{\,\textrm{Sing}\,}}^+\, \mathcal F$ and so by [7, Lemma 1.2.1] there exists an analytic neighbourhood *U* of *z* and a smooth morphism $p:U \rightarrow V$ such that ${{\mathcal {F}}}|_U$ is the foliation induced by *p*. If $v \in V$ is a general closed point, set $L_v := p^{-1}(v)$. Thus, after possibly shrinking again, we may assume that *Z* and *W* are smooth. In particular, *n* is an isomorphism.

Since *W* is not contained in the support of $\Delta$, it follows that for a general closed point $v \in p(Z)$, $L_v$ is not contained in the support of $\Delta$. Since *p* is smooth, there exists $\mu >0$ such that $(L_v, \mu \Delta |_{L_v})$ is log canonical. Thus, Lemma 4.4 implies that $({\mathcal {F}}, \mu \Delta )$ is log canonical in a neighbourhood of $L_v$. Since *v* is general it follows that $({\mathcal {F}}, \mu \Delta )$ is log canonical at the generic point of *Z*. By Lemma 4.3, we have that ${\mathcal {F}}$ is terminal at the generic point of *Z*, and so $({\mathcal {F}}, \frac{\mu }{2}\Delta )$ is log terminal at the generic point of *Z*. By [30, Corollary III.i.4], we have that for any birational morphism $\pi :X' \rightarrow X$, the $\pi$-exceptional locus is $(\pi ^{-1}{\mathcal {F}})$-invariant, and so in fact $({\mathcal {F}}, \frac{\mu }{2} \Delta )$ is terminal at the generic point of *Z*. Thus, (1) follows.

We now prove Item (2). For a general closed point $v \in p(Z)$, $L_v$ is not contained in $\Delta$ and so may apply Lemma 4.4 to see that if $({{\mathcal {F}}},\Delta )$ is log canonical then $(L_v, \Delta \vert _{L_v})$ is log canonical. Let ${{\mathcal {F}}}_{W}$ be the restricted foliation on *W*, whose existence is guaranteed by Proposition-Definition 3.12 Since *W* is smooth, we have that $\textrm{Diff}_W(\mathcal {F},\Delta )|_{L_v}=\Delta |_{L_v}$. Thus, we may again apply Lemma 4.4 to see that $({\mathcal {F}}_W, \textrm{Diff}_W(\mathcal {F},\Delta ))$ is log canonical in a neighbourhood of $L_v$. Since *v* is general we see that $({\mathcal {F}}_W, \textrm{Diff}_W({{\mathcal {F}}},\Delta ))$ is log canonical in a neighbourhood of the generic point of *Z*. $\square$

### Lemma 4.6

Let *X* be a normal variety, let $\mathcal F$ be a rank one foliation on *X* such that $K_{{\mathcal {F}}}$ is ${\mathbb {Q}}$-Cartier. Let $C \subset X$ be a curve which is not $\mathcal {F}$-invariant and is not contained in ${{\,\textrm{Sing}\,}}^+\, {{\mathcal {F}}}$.

Then there exists a birational morphism $p:X' \rightarrow X$ such that if ${{\mathcal {F}}}' := p^{-1}{{\mathcal {F}}}$ then the following hold:

1. $K_{{{\mathcal {F}}}'}+ E = p^*K_{{{\mathcal {F}}}}$ for some *p*-exceptional $\mathbb {Q}$-divisor $E\ge 0$;
2. $p^{-1}$ is an isomorphism at the general point of *C* and if $C'$ is the strict transform of *C* in $X'$ then ${{\mathcal {F}}}'$ is terminal at all points $P \in C'$; and
3. after possibly replacing *X* by an analytic neighbourhood of *C*, there exist a ${{\mathcal {F}}}'$-invariant surface $\Gamma$ containing $C'$.

### Proof

Since *C* is not ${{\mathcal {F}}}$-invariant and *C* is not contained in ${{\,\textrm{Sing}\,}}^+\, {{\mathcal {F}}}$, by Lemma 4.3 we have that ${{\mathcal {F}}}$ is terminal at the generic point of *C*. Let $P_1,\ldots , P_k\in C$ be all the closed points at which ${{\mathcal {F}}}$ is not terminal. Let *H* be a sufficiently general ample divisor on *X* such that $P_1,\dots ,P_k$ are not contained in its support. We may assume that ${\mathcal {O}}_X(mK_{{\mathcal {F}}})\vert _{X{\setminus } H} \cong \mathcal O_{X{\setminus } H}$ where $m>0$ is the Cartier index of $K_{\mathcal F}$. Thus, we may find a finite Galois morphism $\sigma :Y \rightarrow X$ with Galois group *G*, which is quasi-étale outside *H* and such that $\sigma ^*K_{{{\mathcal {F}}}}$ is Cartier around any closed point $Q\in {{\widetilde{C}}}$ such that $\sigma (Q)\in \{P_1,\dots ,P_k\}$. Let ${{\mathcal {G}}} := \sigma ^{-1}{{\mathcal {F}}}$ and let ${{\widetilde{C}}} := \sigma ^{-1}(C)$. Then $K_{{{\mathcal {G}}}}$ is Cartier around any closed point $Q\in {{\widetilde{C}}}$ such that $\sigma (Q)\in \{P_1,\dots ,P_k\}$ and ${{\mathcal {G}}}$ is terminal at all closed points $Q\in {{\widetilde{C}}}$ such that $\sigma (Q)\notin \{P_1,\dots ,P_k\}$.

By [7, Proposition I.2.4], there exists a birational morphism $q:Y' \rightarrow Y$, obtained as a sequence of *G*-equivariant blow ups in ${{\mathcal {G}}}$-invariant points, such that in a neighbourhood of $q^{-1}(\sigma ^{-1}(\{P_1, \dots , P_k\}))$ the strict transform ${{\widetilde{C}}}'$ of ${{\widetilde{C}}}$ in $Y'$ is disjoint from ${{\,\textrm{Sing}\,}}^+ q^{-1}{{\mathcal {G}}}$. Moreover, [7, Proposition I.2.4] implies that $K_{q^{-1}{{\mathcal {G}}}} + E'= q^*K_{{{\mathcal {G}}}}$ where $E' \ge 0$ is *q*-exceptional.

We have that *G* acts on $Y'$ and *q* is *G*-invariant. Thus, if $X' := Y'/G$ then there exists a birational morphism $p:X' \rightarrow X$. It follows by [18, Lemma 3.4] that $K_{{{\mathcal {F}}}'} +E = p^*K_{{{\mathcal {F}}}}$ where ${{\mathcal {F}}}' = p^{-1}{{\mathcal {F}}}$ and $E \ge 0$ is *p*-exceptional (see also [10, Proposition 2.2]). Thus, (1) follows.

Notice that $p^{-1}$ is an isomorphism outside $P_1,\dots ,P_k$ and, in particular, ${{\mathcal {F}}}'$ is terminal at all points in $C'{\setminus } p^{-1}(\{P_1,\dots ,P_k\})$. We now show that ${\mathcal {F}} '$ is terminal at all points of $C'$. By [30, Corollary III.i.4], we have that exceptional divisor over $Y'$ and whose centre is in ${{\widetilde{C}}}' \cap q^{-1}(\sigma ^{-1}(\{P_1, \dots , P_k\}))$, is invariant. Thus, every exceptional divisor over $X'$ and whose centre is in $C' \cap p^{-1}(\{P_1, \dots , P_k\}))$ is also invariant. As in the proof of [16, Lemma 4.3] (see also [30, Corollary III.i.5]), we have that ${{\mathcal {F}}}'$ is terminal at any point in $C'\cap p^{-1}(\{P_1,\dots ,P_k\})$. Thus, (2) follows.

By [7, Proposition I.2.4] there also exists a $q^{-1}{\mathcal {G}}$-invariant surface germ *S* containing $q_*^{-1}{\widetilde{C}}$. We may take $\Gamma = r(S)$ where $r:Y' \rightarrow X'$ is the quotient map and so (3) follows. $\square$

### Lemma 4.7

Let *X* be a normal variety and let $(\mathcal {F}, \Delta )$ be a rank one foliated pair on *X* such that $K_{\mathcal {F}}$ and $\Delta$ are ${\mathbb {Q}}$-Cartier and $\Delta \ge 0$. Let $C \subset {{\,\textrm{Sing}\,}}^+\, {{\mathcal {F}}}$ be a curve and suppose that $({{\mathcal {F}}}, \Delta )$ is log canonical at the generic point of *C*.

Then $$(K_{{{\mathcal {F}}}}+\Delta )\cdot C\ge 0$$.

### Proof

First suppose that $\Delta = 0$. When ${{\mathcal {F}}}$ is Gorenstien this is proven in [7, §4.1], the general case where ${{\mathcal {F}}}$ is only $\mathbb {Q}$-Gorenstein is proven in [28, Fact II.d.3].

Assume now that $\Delta \ne 0$. We claim that *C* is not contained in $\textrm{Supp}\, \Delta$. Supposing the claim we have that $\Delta \cdot C \ge 0$ and by the previous case it follows that $(K_{{{\mathcal {F}}}}+\Delta )\cdot C \ge 0$.

We now prove the claim. We may replace *X* by a neighbourhood of the generic point of *C* and so we may assume that the index one cover associated to $K_{{\mathcal {F}}}$ exists. By [30, Corollary III.i.5] and by the definition of ${{\,\textrm{Sing}\,}}^+\, {\mathcal {F}}$, we may also freely replace *X* by this index one cover, and so we may freely assume that $K_{{\mathcal {F}}}$ is Cartier and so ${\mathcal {F}}$ is generated by a vector field $\partial$. Since $C \subset {{\,\textrm{Sing}\,}}^+\, {\mathcal {F}}$, it follows that *C* is $\mathcal F$-invariant. Thus, [7, Lemma 1.1.3] implies that the blow up $b:X' \rightarrow X$ along *C* extracts a divisor *E* of discrepancy $\le 0$. Since *E* is also $b^{-1}{{\mathcal {F}}}$-invariant it follows that the discrepancy of *E* is in fact $\le -\epsilon (E)$. Since $({\mathcal {F}}, \Delta )$ is log canonical at the generic point of *C*, we may then conclude that *C* is not contained in $\textrm{Supp}\, \Delta$ and our claim follows. $\square$

### Theorem 4.8

Let *X* be a normal projective variety, let $\mathcal {F}$ be a rank one foliation on *X* and let $\Delta \ge 0$ be a ${\mathbb {Q}}$-divisor on *X* such that $K_{{{\mathcal {F}}}}$ and $\Delta$ are ${\mathbb {Q}}$-Cartier.

Then there are ${\mathcal {F}}$-invariant rational curves $C_1,C_2,\dots$ on *X* not contained in ${{{\,\textrm{Sing}\,}}^+ }\, {\mathcal {F}}$ such that

$$\begin{aligned} 0<-(K_{{{\mathcal {F}}}}+\Delta )\cdot C_i\le 2\dim X\end{aligned}$$

and

$$\begin{aligned} \overline{\textrm{NE}}(X)=\overline{\textrm{NE}}(X)_{K_{{{\mathcal {F}}}}+\Delta \ge 0}+Z_{-\infty }+ \sum _i {\mathbb {R}}_+[C_i] \end{aligned}$$

where $Z_{-\infty }\subset \overline{\textrm{NE}}(X)$ is a closed subcone contained in the span of the images of $\overline{\textrm{NE}}(T) \rightarrow \overline{\textrm{NE}}(X)$ of non-log canonical centres $T \subset X$ of $({{\mathcal {F}}}, \Delta )$.

### Proof

Let $R \subset {\overline{NE}}(X)$ be a $(K_{{{\mathcal {F}}}}+\Delta )$-negative exposed extremal ray (e.g. see [34, Definition 6.1]) and let $H_R$ be a supporting hyperplane to *R*. After possibly rescaling $H_R$ we may assume that there exists an ample $\mathbb R$-divisor *H* such that $H_R\sim _{{\mathbb {R}}}K_{{{\mathcal {F}}}}+\Delta +H$. Let *W* be a component of the null locus $\textrm{Null} \, H_R$ of $H_R$ (e.g. see [6, Definition 1.3]) and let $n:S\rightarrow W$ be its normalisation. Thus, $H_R|_S$ is not big and by Nakamaye’s Theorem [6, Theorem 1.4], it follows that *W* is a component of the stable base locus of $H_R-A$ for any sufficiently small ample ${\mathbb {R}}$-divisor *A* on *X*. We want to show that, after possibly replacing *W* by another irreducible component, *R* is contained in the image of $\overline{\textrm{NE}}(W) \rightarrow \overline{\textrm{NE}}(X)$. Indeed, first note that we can take *A* so that $H_R-A$ is a ${\mathbb {Q}}$-divisor. Since $H_R\cdot \xi =0$ for all $\xi$ such that $[\xi ]\in R$, we may find a sequence of classes $[\xi _i]\in \textrm{NE}(X)$ such that $(H_R-A)\cdot \xi _i<0$ and whose limit is a non zero element $[\xi _0]\in R$. Thus, after possibly taking a subsequence and after possibly replacing $\xi _i$ by an irreducible curve, we may assume that each $\xi _i$ is contained in the same connected component of the stable base locus of $H_R-A$. Thus, our claim follows.

By [30, Definition/Summary I.ii.6], the union of all the non-log canonical centres of $({{\mathcal {F}}},\Delta )$ is closed in *X*. If $({{\mathcal {F}}},\Delta )$ is not log canonical at the generic point of *W* then $R\subset Z_{-\infty }$ and we are done. Thus, we may assume that $({{\mathcal {F}}},\Delta )$ is log canonical at the generic point of *W*.

We now distinguish three cases. Suppose first that *W* is contained in ${{\,\textrm{Sing}\,}}^+\, {\mathcal {F}}$. In this case, Lemma 4.7 implies that every curve in *W* which is $(K_{\mathcal F}+\Delta )$-negative is contained in a non-log canonical centre of $({\mathcal {F}}, \Delta )$. Thus, there exists a subvariety $T\subset W$ which is a non-log canonical centre of $({{\mathcal {F}}},\Delta )$ and such that *R* is contained in the image of $\overline{\textrm{NE}}(T) \rightarrow \overline{\textrm{NE}}(X)$ and we may conclude.

Now suppose that *W* is not contained in ${{\,\textrm{Sing}\,}}^+\, {\mathcal {F}}$ and is ${\mathcal {F}}$-invariant. In particular, [7, Lemma I.1.3] implies that *W* is a log canonical centre for ${{\mathcal {F}}}$. Thus, since $({{\mathcal {F}}},\Delta )$ is log canonical at the generic point of *W*, it follows that *W* is not contained in the support of $\Delta$. Let ${{\mathcal {F}}}_{S}$ be the restricted foliation on *S*, whose existence is guaranteed by Proposition-Definition 3.12 and let $\Delta _{S} := \textrm{Diff}_S({{\mathcal {F}}},\Delta )$. Since $H_R|_S$ is not big, we may apply [34, Corollary 2.28] to conclude that *S* is covered by $(K_{{{\mathcal {F}}}_S}+\Delta _S)$-negative rational curves *C* tangent to ${{\mathcal {F}}}$ which span *R* and such that

$$\begin{aligned}0<-(K_{{{\mathcal {F}}}_{S}}+\Delta _{S})\cdot C \le 2\dim S, \end{aligned}$$

(e.g. see the proof of [34, Theorem 6.3] for more details). Thus, we may easily conclude.

Finally suppose that *W* is not contained in ${{{\,\textrm{Sing}\,}}}^+\, \mathcal F$ and is not ${\mathcal {F}}$-invariant. We show that this case does not in fact occur by showing that it leads to a contradiction. In this case, *W* is a proper subvariety of *X* and $H_R$ is big. Let $0<\epsilon \ll 1$ be a rational number such that $H_R-\epsilon H$ is big and $\textrm{Null}\, H_R$ coincides with the stable base locus of $H_R-\epsilon H$. Let $G\sim _{{\mathbb {Q}}} H_R-\epsilon H$ be an effective ${\mathbb {Q}}$-divisor. Since $H_R|_S$ is not big and *H* is ample, there exists a curve $C\subset S$ passing through the general point of *S* and such that $G\cdot C<0$. Moreover, we have

$$\begin{aligned} (K_{{\mathcal {F}}}+ \Delta )\cdot C = (H_R-H)\cdot C=\left( G - (1-\epsilon )H\right) \cdot C<0. \end{aligned}$$

Note that, from now on, we no longer require $K_{{{\mathcal {F}}}}$ and $\Delta$ to be ${\mathbb {Q}}$-Cartier, but just that $K_{\mathcal {F}}+\Delta$ is ${\mathbb {Q}}$-Cartier. Thus, we may replace *X* by a model guaranteed to exist by Lemma 4.6, and so we may find a germ of a ${{\mathcal {F}}}$-invariant surface $\Gamma$ containing *C*.

Since *W* is not ${{\mathcal {F}}}$-invariant and $\Gamma$ intersect the general point of *W*, it follows that $\Gamma$ is not contained in *W*. In particular, since *W* is a component of the stable base locus of $H_R-\epsilon H$, after possibly replacing *G* by an effective ${\mathbb {Q}}$-divisor $G'$ which is ${\mathbb {Q}}$-linearly equivalent to *G*, we may assume that $\Gamma$ is not contained in the support of *G*. Let $\nu :Y\rightarrow \Gamma$ be the normalisation. By Proposition-Definition 3.12, if ${{\mathcal {F}}}_Y$ is the foliation induced on *Y* then we may write

$$\begin{aligned} (K_{{\mathcal {F}}}+ \Delta )\vert _Y \sim _{{\mathbb {Q}}} K_{\mathcal F_Y}+\Delta _Y \end{aligned}$$

where $\Delta _Y := \textrm{Diff}_Y({{\mathcal {F}}},\Delta )$. By Lemma 4.5, we have that $({\mathcal {F}}_Y, \Delta _Y)$ is log canonical at the generic point of *C* and, in particular, $m_C\Delta _Y\le 1$. Let $t\ge 0$ such that $\Delta _Y+tC=\Theta +C$ where $\Theta \ge 0$ is a ${\mathbb {Q}}$-divisor whose support does not contain *C*. Let $\mu >0$ be a rational number such that $m_C(\mu G|_Y)=1$. Then, considering *C* as a curve in *Y*, we have $C^2\le C\cdot \mu G|_Y<0$ and so

$$\begin{aligned} (K_{{{\mathcal {F}}}_Y}+\Theta +C)\cdot C=(K_{{{\mathcal {F}}}}+\Delta )\cdot C+tC^2<0. \end{aligned}$$

On the other hand, by [34, Proposition 3.4] (note that the proposition is stated to hold for projective varieties, but it works equally well in our context) and since the restricted foliation on *C* is the foliation by points, we have that $(K_{{{\mathcal {F}}}_Y}+\Theta +C)\cdot C\ge 0$, a contradiction. Thus, our result follows. $\square$

Using the same methods as in the last case of the proof above, we have the following:

### Corollary 4.9

Let *X* be a normal projective variety, let $\mathcal {F}$ be a rank one foliation on *X* and let $\Delta \ge 0$ be a ${\mathbb {Q}}$-Cartier divisor such that $K_{{{\mathcal {F}}}}$ and $\Delta$ are ${\mathbb {Q}}$-Cartier. Assume that $K_{{{\mathcal {F}}}}+\Delta$ is nef and $({{\mathcal {F}}},\Delta )$ is log canonical away from a finite set of closed points.

Then $\textrm{Null} \, (K_{{{\mathcal {F}}}}+\Delta )$ is a union of $\mathcal {F}$-invariant subvarieties, and subvarieties contained in ${{\,\textrm{Sing}\,}}^+\, {\mathcal {F}}$.

In [13, Theorem 1.2] a dynamical characterisation of ample line bundles on smooth surfaces was provided. As a consequence of the above theorem we are able to extend this to higher dimensions:

### Corollary 4.10

Let *X* be a normal projective variety and let *L* be a ${\mathbb {Q}}$-Cartier divisor. Suppose that

1. $$L^{\dim X} \ne 0$$;
2. for some $q >0$ there exists a rank one foliation ${\mathcal {F}}$ such that $K_{{{\mathcal {F}}}}$ is ${\mathbb {Q}}$-Cartier and $K_{{\mathcal {F}}} \equiv qL$; and
3. $\dim {{\,\textrm{Sing}\,}}^+ \, {\mathcal {F}}\le 0$ and ${\mathcal {F}}$ admits no invariant positive dimensional subvarieties.

Then *L* is ample.

### Proof

By (3) and by Theorem 4.8, it follows that *L* is nef and so by (1) we have that $L^{\dim X}>0$, and hence *L* is big. By Lemma 4.3, ${{\mathcal {F}}}$ is log canonical outside ${{\,\textrm{Sing}\,}}^+ \, {\mathcal {F}}$ and by Corollary 4.9 it follows that $\textrm{Null}~ K_{{\mathcal {F}}} = \emptyset$ and so by the Nakai–Moishezon criterion for ampleness we see that $K_{{\mathcal {F}}}$ is ample. $\square$

## Family of leaves of an algebraically integrable foliation

Let *X* be a normal projective variety and let ${\mathcal {F}}$ be an algebraically integrable foliation on *X*. The following construction follows from [2, Lemma 3.2]:

### Construction 5.1

There exists a closed subvariety $Z \subset \textrm{Chow}(X)$ whose general point parametrises the closure of a leaf of *X*. Let $p_X:X\times Z \rightarrow X$ and $p_Z :X \times Z \rightarrow Z$ be the two projections.

If we let $U \subset X \times Z$ to be the universal cycle and $n:{\widehat{X}} \rightarrow U$ be the normalisation, then we have

1. a birational morphism $\beta :{\widehat{X}} \rightarrow X$ where $\beta = p_X\vert _{U} \circ n$;
2. an equidimensional contraction $f:{\widehat{X}} \rightarrow {\widehat{Z}}$ where ${\widehat{Z}}$ is the normalisation of *Z*; and
3. a foliation $\widehat{{\mathcal {F}}} := \beta ^{-1}{\mathcal {F}}$, which is induced by *f*.

### Lemma 5.2

Set up as above. Suppose that $K_{{\mathcal {F}}}$ is ${\mathbb {Q}}$-Cartier.

Then we may write $K_{\widehat{{\mathcal {F}}}}+F \sim _{{\mathbb {Q}}} \beta ^*K_{{\mathcal {F}}}$ where $F \ge 0$ is $\beta$-exceptional. Moreover, if *E* is a $\beta$-exceptional divisor which is not $\widehat{{\mathcal {F}}}$-invariant then *E* is contained in $\textrm{Supp}\, F$.

### Proof

This is [18, Section 3.6] and [18, Proposition 4.17]. $\square$

### Lemma 5.3

Let $f:X \rightarrow Z$ be an equidimesional contraction between normal varieties and let ${\mathcal {F}}$ and ${\mathcal {G}}$ be foliations on *X* and *Z* respectively such that ${\mathcal {F}} = f^{-1}{\mathcal {G}}$. Let ${\mathcal {H}}$ be the foliation induced by $X \rightarrow Z$.

Then

$$\begin{aligned} K_{{\mathcal {H}}} = (K_{{\mathcal {F}}}-f^*K_{{\mathcal {G}}})-R(f)_{\mathrm{n-inv}} \end{aligned}$$

where $R(f)_{\mathrm{n-inv}}$ denotes the part of the ramification divisor *R*(*f*) of *f* which is not ${{\mathcal {F}}}$-invariant.

### Proof

The desired equality may be checked away from a subset of codimension at least two and so, without loss of generality, we may assume that $X, Z, {\mathcal {H}}, {\mathcal {F}}$ and ${\mathcal {G}}$ are smooth. We may then apply Formula (2.1) in [16, §2.9] to conclude. $\square$

### Theorem 5.4

Let *X* be a ${\mathbb {Q}}$-factorial klt projective variety and let ${\mathcal {F}}$ be a foliation on *X*. Let ${\mathcal {H}}$ be the algebraic part of ${\mathcal {F}}$ and let $\beta :{\widehat{X}} \rightarrow X$ be the morphism guaranteed to exist by Construction 5.1.

Then $a(E,{\mathcal {H}})\ge a(E,{\mathcal {F}})$ for any $\beta$-exceptional prime divisor *E* which is not $\beta ^{-1}\mathcal {H}$-invariant. In particular, if ${{\mathcal {F}}}$ admits canonical singularities, then ${\mathcal {H}}$ is induced by an almost holomorphic map.

### Proof

Using the same notation as in Construction 5.1, note that ${{\mathcal {H}}}$ is induced by the rational map $f \circ \beta ^{-1} :X\dashrightarrow {\widehat{Z}}$. Let $\widehat{{{\mathcal {F}}}} := \beta ^{-1}{{{\mathcal {F}}}}$ and let $\widehat{{{\mathcal {H}}}}:= \beta ^{-1}{{{\mathcal {H}}}}$. By definition, there exists a purely transcendental foliation $\widehat{{\mathcal {G}}}$ on ${\widehat{Z}}$ such that $\widehat{{\mathcal {F}}} = {\widehat{f}}^{-1}\widehat{\mathcal G}$.

We may write

$$\begin{aligned} K_{\widehat{{\mathcal {F}}}} +F= \beta ^*K_{{\mathcal {F}}} \end{aligned}$$

and

$$\begin{aligned} K_{\widehat{{\mathcal {H}}}} + G= \beta ^*K_{{\mathcal {H}}} \end{aligned}$$

where *F*, *G* are $\beta$-exceptional and by Lemma 5.2, we have that $G\ge 0$.

By Lemma 5.3 we have

$$\begin{aligned} K_{\widehat{{\mathcal {H}}}}+{{{\widehat{f}}}}^*K_{\widehat{\mathcal G}}+R({{\widehat{f}}})_{\mathrm{n-inv}} = K_{\widehat{{{\mathcal {F}}}}}, \end{aligned}$$

where $R({{\widehat{f}}})_{\mathrm{n-inv}}$ denotes the part of $R({{\widehat{f}}})$ which is not $\widehat{ {{\mathcal {F}}}}$-invariant. Thus,

$$\begin{aligned} G-F \sim _{{\mathbb {Q}},\beta } {{{\widehat{f}}}}^*K_{\widehat{{\mathcal {G}}}}+ R({{\widehat{f}}})_{\mathrm{n-inv}}. \end{aligned}$$

Notice that $K_{\widehat{{\mathcal {G}}}}$ is pseudo-effective since $\widehat{{\mathcal {G}}}$ is purely transcendental (see [9, Corollary 4.13]). Let *A* be an ample divisor on ${\widehat{X}}$ and let $\Sigma$ be a $\beta$-exceptional prime divisor. Since $G-F$ is $\beta$-exceptional and ${{{\widehat{f}}}}^*K_{\widehat{{\mathcal {G}}}}+ R({{\widehat{f}}})_{\mathrm{n-inv}}$ is pseudo-effective, the negativity lemma (cf. [25, Lemma 3.39]) implies that if $m_\Sigma (G-F)>0$ then $\Sigma$ is contained in the stable base locus of ${{{\widehat{f}}}}^*K_{\widehat{{\mathcal {G}}}}+ R(\widehat{f})_{\mathrm{n-inv}}+tA$ for any sufficiently small rational number $t>0$. In particular, $\Sigma$ is $\widehat{{{\mathcal {H}}}}$-invariant.

We deduce from this that for any $\beta$-exceptional prime divisor *E* which is not $\widehat{{{\mathcal {H}}}}$-invariant we have $a(E, \mathcal H) \ge a(E, {\mathcal {F}})$.

We now prove our second claim. Assume by contradiction that ${{\mathcal {F}}}$ admits canonical singularities but *f* is not almost holomorphic. Then there exists a $\beta$-exceptional divisor *E* which dominates ${\overline{Z}}$. By Lemma 5.2, we have that *E* is contained in the support of *G*. Thus, $a(E,{\mathcal {F}})\le a(E,{\mathcal {H}})<0$, a contradiction. $\square$

### Corollary 5.5

Let *X* be a smooth projective variety and let ${\mathcal {F}}$ be a foliation on *X* with canonical singularities. Suppose that $-K_{{\mathcal {F}}}$ is nef and is not numerically trivial.

Then the algebraic part of ${\mathcal {F}}$ is induced by an equidimensional fibration.

### Proof

By [9, Corollary 4.13], the algebraic part ${\mathcal {H}}$ of ${\mathcal {F}}$ is non-trivial, and by Theorem 5.4, ${\mathcal {H}}$ is induced by an almost holomorphic map $f:X\dashrightarrow Z$. Following the proof of [17, Claim 4.3] we see that $K_{{\mathcal {F}}} \equiv K_{{\mathcal {H}}}$ and, in particular $-K_{{{\mathcal {H}}}}$ is nef. The result then follows by [31, Corollary 1.4]. $\square$

## Data Availability

The authors declare no data were used in the paper.
